# Four-component reaction of cyclic amines, 2-aminobenzothiazole, aromatic aldehydes and acetylenedicarboxylate

**DOI:** 10.3762/bjoc.9.330

**Published:** 2013-12-27

**Authors:** Hong Gao, Jing Sun, Chao-Guo Yan

**Affiliations:** 1 College of Chemistry & Chemical Engineering, Yangzhou University, Yangzhou 225002, China

**Keywords:** benzothiazole, domino reaction, electron-deficient alkyne, multicomponent reaction, pyrrolidinone

## Abstract

The four-component reaction of 2-aminobenzothiazole, aromatic aldehydes, acetylenedicarboxylate and piperidine or pyrrolidine in ethanol afforded the functionalized 2-pyrrolidinones containing both benzothiazolyl and piperidinyl (or pyrrolidinyl) units in good yields. On the other hand, the similar four-component reactions resulted in the functionalized morpholinium or piperidinium 2-pyrrolidinon-3-olates in the presence of *p*-toluenesulfonic acid.

## Introduction

Over fifty years ago, Huisgen firstly described the addition reactions of nitrogen-containing heterocycles to electron-deficient alkynes to form 1,4-dipolar intermediates, which can reacted sequentially with other reagents to give cycloaddition products [1–2]. From then on much developments on the chemistry of Huisgen 1,4-dipoles have been achieved [3–4]. In the past few years, Huisgen 1,4-dipoles have been recognized as key components for designing practical multicomponent reactions and domino reactions, mainly due to their easy generation and versatile reactivity [5–10]. On the other hand, the similar reactive Huisgen 1,4-dipoles derived from the addition of primary or secondary amines to electron-deficient alkynes also provided many elegant procedures for the synthesis of various nitrogen-containing heterocycles [11–16]. In this hot research field, we also successfully developed a series of domino reactions containing primary amine, electron-deficient alkynes and the other components, and found several efficient synthetic protocols for versatile heterocycles and spiro compounds by using the in situ generated Huisgen 1,4-dipoles [17–24]. During these research works, we noticed that even through the cyclic secondary amines such as pyrrolidine, piperidine and morpholine also reacted with electron-deficient alkynes to give the Huisgen 1,4-dipoles very fast and in nearly quantitative yields [25–26]. But until now it seems that this kind of easily generated Huisgen 1,4-dipoles have not been utilized for the design of domino reactions. In continuation of our efforts to explore the practical applications of Huisgen 1,4-dipoles for the synthesis of a versatile heterocyclic system, herein we wish to report the interesting results of the four-component reaction of secondary cyclic amines, acetylenedicarboxylate, 2-aminobenzothiazole and aromatic aldehydes and the efficient synthesis of the complex 2-pyrrolidinones containing both benzothiazolyl and piperidinyl (or pyrrolidinyl) units.

## Results and Discussion

Initially, we set out to investigate the reaction conditions by using piperidine to react with dimethyl acetylenedicarboxylate to give the expected β-enamino ester. It is interesting to find that the reaction of piperidine with acetylenedicarboxylate in ethanol at room temperature proceeded very quickly and could be finished to give the expected β-enamino ester in less than twenty minutes [27], while the reaction of normal primary arylamine with acetylenedicarboxylate or propiolate in ethanol at room temperature usually needed more than one day [28]. Thus we chose to employ a one-pot multicomponent reaction procedure to investigate our reaction. A mixture of dimethyl acetylenedicarboxylate, benzaldehyde, 2-aminobenzothiazole and excess piperidine in ethanol was stirred at room temperature for about twenty minutes and then was heated at 50–60 °C for about 48 hours. In this reaction the excess piperidine acted as base catalyst. After work-up, the expected polyfunctionalized 2-pyrrolidinone **1a** was obtained in good yield (Table 1, entry 1). Under similar reaction conditions, various aromatic aldehydes were utilized in the reaction to give the polyfunctionalized 2-pyrrolidinone **1b**–**1f** (Table 1, entries 2–6) in 53–72% yields, respectively. The four-component reaction containing diethyl acetylenedicarboxylate also successfully afforded the expected 2-pyrrolidinone **1g** in 63% (Table 1, entry 7).

**Table 1:** Synthesis of pyrrolidinones **1a**–**1n** via four-component reactions^a^.

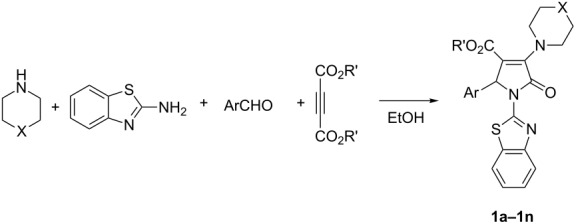					

Entry	Compd	X	R’	Ar	Yield^b^ (%)

1	**1a**	CH_2_	CH_3_	C_6_H_5_	58
2	**1b**	CH_2_	CH_3_	*m*-CH_3_C_6_H_4_	72
3	**1c**	CH_2_	CH_3_	*p*-CH(CH_3_)_2_C_6_H_4_	55
4	**1d**	CH_2_	CH_3_	*p*-ClC_6_H_4_	53
5	**1e**	CH_2_	CH_3_	*m*-ClC_6_H_4_	67
6	**1f**	CH_2_	CH_3_	*m*-NO_2_C_6_H_4_	70
7	**1g**	CH_2_	CH_2_CH_3_	*p*-CH_3_C_6_H_4_	63
8	**1h**	O	CH_3_	C_6_H_5_	66^c^
9	**1i**	O	CH_3_	*p-*CH_3_OC_6_H_4_	55^c^
10	**1j**	O	CH_3_	*p*-ClC_6_H_4_	58^c^
11	**1k**	O	CH_3_	*m*-ClC_6_H_4_	63^c^
12	**1l**	O	CH_3_	*m*-NO_2_C_6_H_4_	68^c^
13	**1m**	O	CH_3_	*p*-NO_2_C_6_H_4_	52^c^
14	**1n**	O	CH_3_	*p-*CH_3_OC_6_H_4_	10^c^

^a^Reaction conditions: 2-aminobenzothiazole (2.0 mmol), acetylenedicarboxylate (2.0 mmol), aromatic aldehyde (2.0 mmol), piperidine (3.0 mmol) in EtOH (10.0 mL), rt, 20 min, 50–60 °C, 48 h; ^b^Isolated yield; ^c^Pyrrolidine or morpholine (2.0 mmol) and DABCO (0.5 mmol) were used.

**Figure 1:**
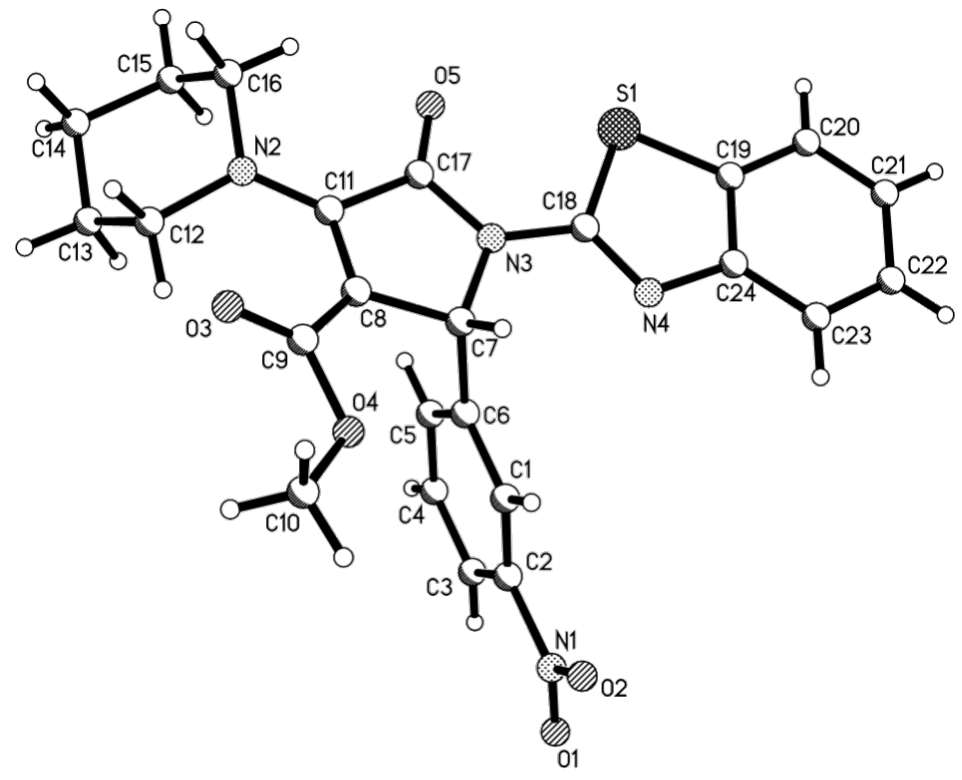
Molecular structure of compound **1f**.

In view of the success of the above reaction, we explored the scope of this promising reaction by varying the structure of the secondary cyclic amines. When excess pyrrolidine was used in the reaction by using the above reaction procedure, it was surprising to find that only very low yields of 3-(pyrrolidin-1-yl)-2-pyrrolidinones were produced. After carefully optimizing the reaction conditions, we were pleased to find that the expected 3-(pyrrolidin-1-yl)-2-pyrrolidinones **1h**–**1m** could be prepared in the satisfactory yields by adding the stronger base DABCO into the reaction as base catalyst (Table 1, entries 8–13). Another common cyclic amine morpholine still gave a very low yield of the desired 3-morpholinyl-2-pyrrolidinone **1n** (Table 1, entry 14). It is known that pyrrolidine (p*K*_b_* =* 2.73) and piperidine (p*K*_b_ = 2.88) have near similar basicity, while morpholine has a relative weak basicity (p*K*_b_* =* 5.64). At present, the exact reason for the different reactivity of piperidine, pyrrolidine and morpholine in this reaction is not very clear. The structures of the prepared 2-pyrrolidinones **1a**–**1n** were fully characterized by ^1^H and ^13^C NMR, HRMS, IR spectra, and were further confirmed by single crystal structure determination of compound **1f** (Figure 1). In ^1^H NMR spectra of compounds **1a**–**1n**, the proton at the 5-position of the newly-formed 2-pyrrolidonyl ring usually displays a singlet at about 6.15 ppm. The piperidin-1yl or pyrrolidin-1-yl groups usually show two or three characteristic mixed peaks.

**Figure 2:**
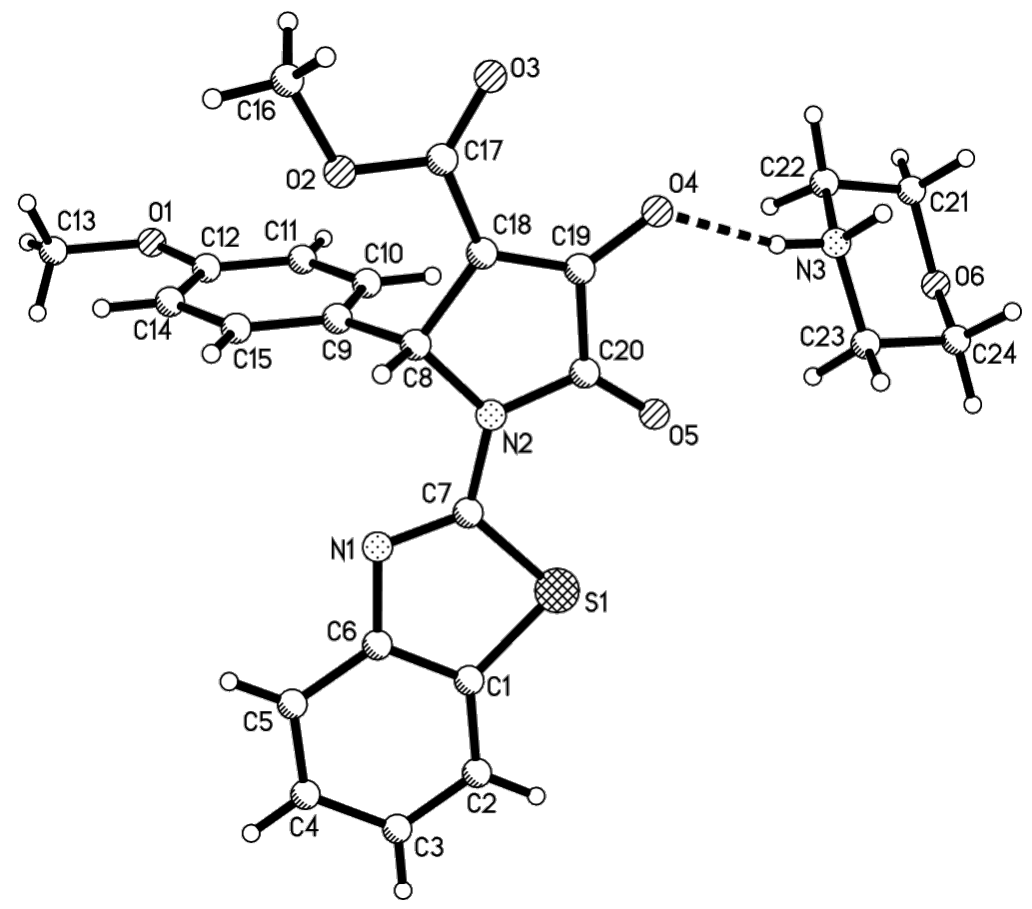
Molecular structure of compound **2a**.

**Figure 3:**
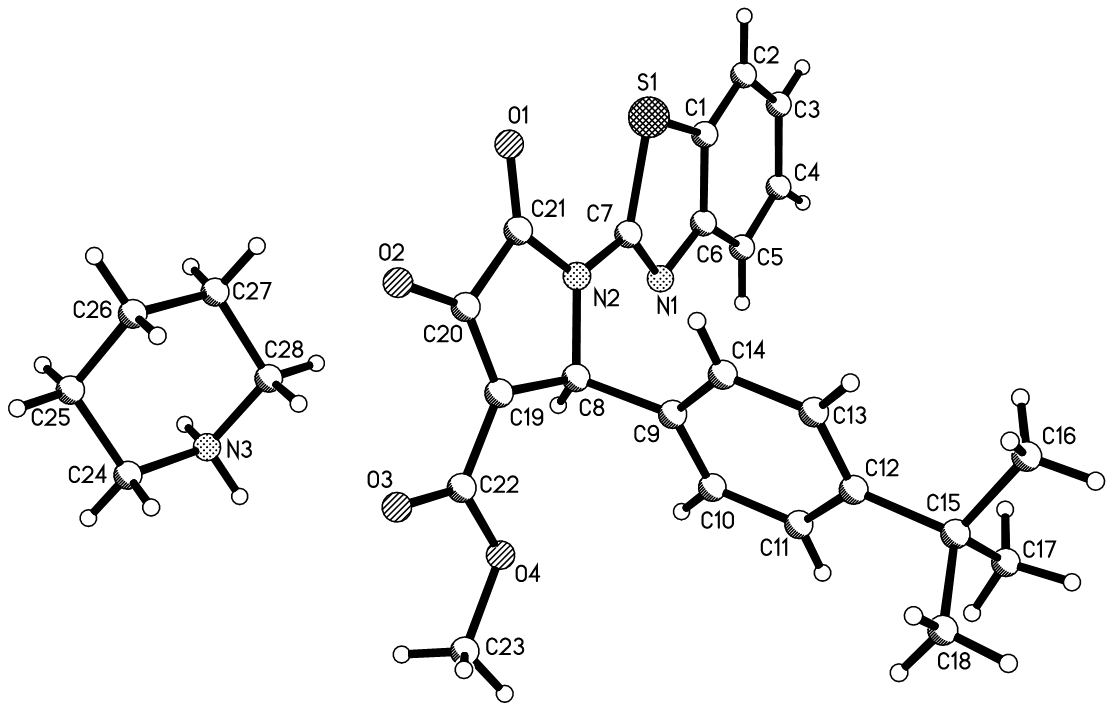
Molecular structure of compound **2h**.

Two years ago, we reported that a *p*-toluenesulfonic acid-catalyzed three-component reaction of arylamine, aromatic aldehyde and acetylenedicarboxylate afforded 3-hydroxy-2-pyrrolidinone as main product [28]. In order to improve the reactivity of morpholine in this four-component reaction, *p*-toluenesulfonic acid was added in the four-component reaction of morpholine, *p-*methoxybenzaldehyde, 2-aminobenzothiazole and dimethyl acetylenedicarboxylate. After work-up, we found that the reaction afforded unexpected morpholinium 2-pyrrolidinon-3-olate **2a** in 75% yield (Table 2, entry 1). Under similar conditions, the reactions with other aromatic aldehydes also gave the morpholinium 2-pyrrolidinon-3-olates **2b**–**2e** (Table 2, entries 2–5) in 65–87% yields, respectively. The formation of morpholinium 2-pyrrolidinon-3-olates **2a**–**2e** clearly indicated that the reaction initially gave the expected 3-hydroxy-2-pyrrolidinone, which in turn converted to enolate by deprotonation of basic morpholine. This result also showed that this four-component reaction has an interesting molecular diversity in basic or acidic solution. We also utilized piperidine in this acid-catalyzed four component reaction and obtained the corresponding piperidinium 2-pyrrolidinon-3-olates **2f**–**2h** in good yields (Table 2, entries 6–8). The prepared piperidinium and morpholinium 2-pyrrolidinon-3-olates **2a**–**2h** are very stable compounds. Their structures were fully characterized by ^1^H and ^13^C NMR, HRMS, IR spectra, and were also confirmed by single crystal structure determination of compounds **2a** (Figure 2) and **2h** (Figure 3).

**Table 2:** Synthesis of pyrrolidinones **2a**–**2h** via four-component reactions^a^.

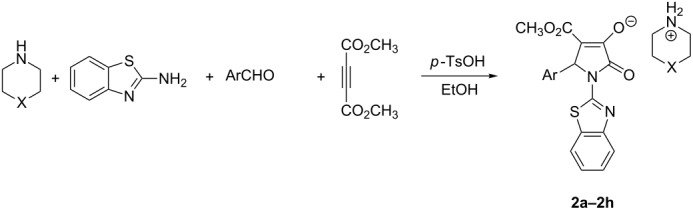				

Entry	Compd	X	Ar	Yield^b^ (%)

1	**2a**	O	*p*-CH_3_OC_6_H_4_	75^c^
2	**2b**	O	*p*-CH(CH_3_)_2_C_6_H_4_	73
3	**2c**	O	*p*-CH_3_C_6_H_4_	65
4	**2d**	O	C_6_H_5_	87
5	**2e**	O	*m*-NO_2_C_6_H_4_	79
6	**2f**	CH_2_	*p*-CH_3_C_6_H_4_	75
7	**2g**	CH_2_	*m*-CH_3_C_6_H_4_	72
8	**2h**	CH_2_	*p*-(CH_3_)_3_CC_6_H_4_	80

^a^Reaction condition: 2-aminobenzothiazole (2.0 mmol), acetylenedicarboxylate; (2.0 mmol), aromatic aldehyde (2.0 mmol), piperidine or piperidine (2.0 mmol), *p*-TsOH (0.5 mmol), in EtOH (10.0 mL), rt, 20 min., 50–60 °C 48 h; ^b^Isolated yield.

**Scheme 1:**
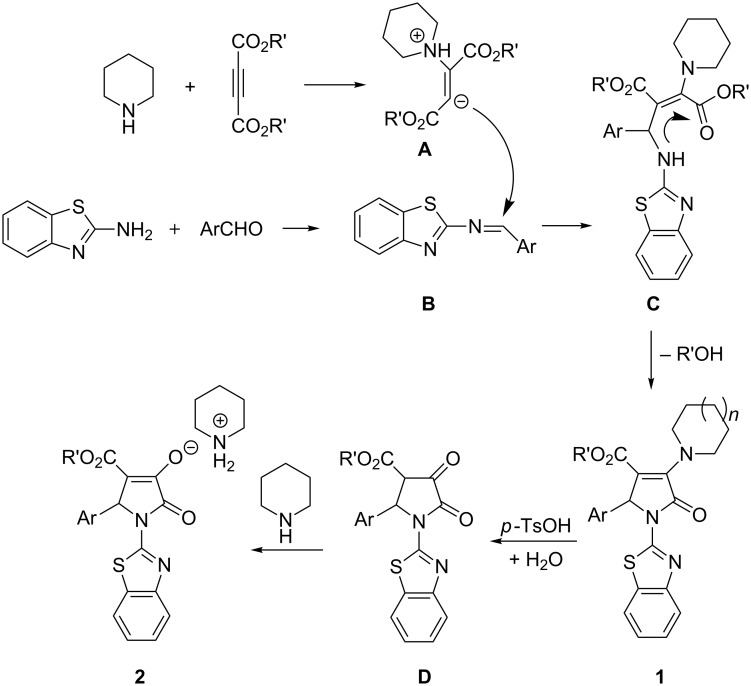
The proposed reaction mechanism for the four-component reaction.

A plausible reaction mechanism for this four-component reaction both in basic media and in acid solution was proposed based on the previous reported similar reactions (Scheme 1) [29–34]. At first, piperidine adds to acetylenedicarboxylate to give 1,3-dipolar intermediate **A**. In the meantime, the condensation of the aromatic aldehyde with 2-aminobenzothiazole affords an aldimine **B**. Secondly, the nucleophilic addition of 1,3-dipole intermediate **A** to aldimine **B** gives an addition intermediate **C**. Thirdly, the intramolecular nucleophilic attack of the amino group to the carbonyl group produces the polyfunctionalized 2-pyrrolidinone **1**. There is one reactive enamine unit in the obtained 2-pyrrolidinone **1**. Under the catalysis of *p*-toluenesulfonic acid, the enamine moiety in 2-pyrrolidinone **1** was easily hydrolyzed to yield a 2, 3-pyrrolidinedione (**D**) and piperidine. Then 2,3-pyrrolidinedione **D** transforms to the more stable enol-form through the keto–enol tautomerism. Because the enol connects to both ester and amide groups, it has much stronger acidity and is deprotonated by piperidine in the solution to give the piperidinium 2-pyrrolidinon-3-olate **2** as the final product.

## Conclusion

In summary, we have successfully developed a four-component reaction of an aromatic aldehyde, 2-aminobenzothiazole, secondary cyclic amines and acetylenedicarboxylate in basic or acidic soution. This four-component reaction provides a convenient procedure for the preparation of the mixed heterocyclic compounds containing units of benzothiazole, piperidine and 2-pyrrolidinone in satisfactory yields. The range of substrates and the reaction mechanism for this reaction were briefly discussed. This convenient synthetic reaction might be potentially used for complex heterocyclic systems in synthetic and medicinal chemistry.

## Experimental

**Reagents and apparatus:** Melting points were taken on a hot-plate microscope apparatus. IR spectra were obtained on a Bruker Tensor 27 spectrometer (KBr disc). NMR spectra were recorded with a Bruker AV-600 spectrometer with DMSO-*d*_6_ as solvent and TMS as internal standard (600 and 150 MHz for ^1^H and ^13^C NMR spectra, respectively). HRMS were measured at UHR-TOF maXis instrument. X-ray data were collected on a Bruker Smart APEX-2 diffractometer. 2-Aminobenzothiazole, dimethyl or diethyl acetylenedicarboxylate, aromatic aldehyde and other reagents are commercial reagents and used as received. Solvents were purified by standard techniques. All reactions were monitored by TLC.

**General procedure for the preparation of the functionalized 2-pyrrolidinones 1a–1n as a one-pot four-component reaction**: A mixture of 2-aminobenzothiazole (2.0 mmol), acetylenedicarboxylate (2.0 mmol), aromatic aldehyde (2.0 mmol), piperidine (3.0 mmol) (in cases of pyrrolidine or morpholine was used in the reaction, pyrrolidine or morpholine (2.0 mmol), DABCO (0.5 mmol)) in ethanol (10.0 mL) was stirred at room temperature for about twenty minutes and then was heated at about 50–60 °C for about two days. After cooling to room temperature, the resulting precipitates were collected by filtration and washed with cold ethanol to give the crude product, which was recrystallized in ethanol to give the pure products **1a**–**1n** for analysis.

**General procedure for the preparation of functionalized 2-pyrrolidinon-3-olates 2a–2h as a one-pot reaction:** A mixture of 2-aminobenzothiazole (2.0 mmol), acetylenedicarboxylate (2.0 mmol), aromatic aldehyde (2.0 mmol), morpholine or piperidine (3.0 mmol) and *p-*toluenesulfonic acid (0.5 mmol) in ethanol (10.0 mL) was stirred at room temperature for about twenty minutes and then was heated at about 50–60 °C for about two days. After cooling to room temperature, the resulting precipitates were collected by filtration and washed with cold ethanol to give the products **2a**–**2h**.

**X-ray crystallographic data:** Single crystal data for compounds **1f** (CCDC 950634), **2a** (CCDC 950635) and **2h** (CCDC 952039) have been deposited in the Cambridge Crystallographic Data Center. These data can be obtained free of charge via http://www.ccdc.ac.ck./data_request/cif .

## Supporting Information

File 1 Analytical data and ^1^H and ^13^C NMR spectra.

## References

[1] Huisgen R, Morikawa M, Herbig K, Brunn E (1967). Chem Ber.

[2] Huisgen R (1968). Z Chem.

[3] Nair V, Rajesh C, Vinod A U, Bindu S, Sreekanth A R, Mathen J S, Balagopal L (2003). Acc Chem Res.

[4] Nair V, Menon R S, Sreekanth A, Abhilash N, Biju A T (2006). Acc Chem Res.

[5] Kielland N, Lavilla R (2010). Top Heterocycl Chem.

[6] Shaabani A, Maleki A, Rezayan A H, Sarvary A (2011). Mol Diversity.

[7] Nair V, Devipriya S, Suresh E (2008). Tetrahedron.

[8] Nair V, Devipriya S, Suresh E (2007). Tetrahedron Lett.

[9] Yavari I, Mirzaei A, Moradi L, Khalili G (2010). Tetrahedron Lett.

[10] Yavari I, Seyfi S, Hossaini Z (2010). Tetrahedron Lett.

[11] Yadav J S, Reddy B V S, Yadav N N, Gupta M K, Sridhar B (2008). J Org Chem.

[12] Ding H, Zhang Y, Bian M, Yao W, Ma C (2008). J Org Chem.

[13] Srikrishna A, Sridharan M, Prasad K R (2010). Tetrahedron.

[14] Bezenšek J, Koleša T, Grošelj U, Wagger J, Stare K, Meden A, Svete J, Stanovnik B (2010). Tetrahedron Lett.

[15] Liu W, Jiang H, Huang L (2010). Org Lett.

[16] Yang J, Wang C, Xie X, Li H, Li Y (2010). Eur J Org Chem.

[17] Sun J, Xia E-Y, Wu Q, Yan C-G (2010). Org Lett.

[18] Sun J, Xia E-Y, Wu Q, Yan C-G (2011). ACS Comb Sci.

[19] Sun J, Sun Y, Gao H, Yan C-G (2011). Eur J Org Chem.

[20] Sun J, Sun Y, Xia E-Y, Yan C-G (2011). ACS Comb Sci.

[21] Sun J, Sun Y, Gong H, Xie Y-J, Yan C-G (2012). Org Lett.

[22] Han Y, Sun Y, Sun J, Yan C-G (2012). Tetrahedron.

[23] Han Y, Wu Q, Sun J, Yan C-G (2012). Tetrahedron.

[24] Sun J, Sun Y, Gao H, Yan C-G (2012). Eur J Org Chem.

[25] Schmidt R R, Kast J, Speer H (1983). Synthesis.

[26] Ziyaei-Halimehjani A, Saidi M R (2008). Tetrahedron Lett.

[27] Sun J, Gao H, Wu Q, Yan C-G (2012). Beilstein J Org Chem.

[28] Sun J, Wu Q, Xia E-Y, Yan C-G (2011). Eur J Org Chem.

[29] Zhu Q, Jiang H, Li J, Liu S, Xia C, Zhang M (2009). J Comb Chem.

[30] Khan A T, Ghosh A, Musawwer M M (2012). Tetrahedron Lett.

[31] Rana S, Brown M, Dutta A, Bhaumik A, Mukhopadhyay C (2013). Tetrahedron Lett.

[32] Ramesh K, Murthy S N, Karnakar K, Nageswar Y V D (2011). Tetrahedron Lett.

[33] Gao H, Sun J, Yan G-C (2013). Tetrahedron.

[34] Sarkar R, Mukhopadhyay C (2013). Tetrahedron Lett.

