# Supplementary material for: Four-component reaction of cyclic amines, 2-aminobenzothiazole, aromatic aldehydes and acetylenedicarboxylate
Source: Beilstein J Org Chem. 2013 Dec 27;9:2934–9. doi: 10.3762/bjoc.9.330 (PMC3896266; doi:10.3762/bjoc.9.330)

Supporting Information

for

Four-component reaction of cyclic amines, 2-aminobenzothiazole, aromatic aldehydes and acetylenedicarboxylate

Hong Gao, Jing Sun, Chao-Guo Yan*

College of Chemistry & Chemical Engineering, Yangzhou University, Yangzhou 225002, China

Email: Chao-Guo Yan – cgyan@yzu.edu.cn

* Corresponding author

Analytical data and ^{1}H and ^{13}C NMR spectra.

Methyl-1-(benzo[*d*]thiazol-2-yl)-5-oxo-2-phenyl-4-(piperidin-1-yl)-2,5-dihydro-1*H*-pyrrole-3-carboxylate (**1a**): white solid, 58%, m.p. 207.6~207.9℃; ^{1}H NMR (600 MHz, $CDCl_3$) δ: 7.75 (s, 1H, ArH), 7.71 (s, 1H, ArH), 7.39 (s, 2H, ArH), 7.35 (s, 1H, ArH), 7.19 (s, 4H, ArH), 6.14 (s, 1H, CH), 3.67 (s, 4H, CH_2), 3.59 (s, 3H, OCH_3), 1.76~1.65 (m, 6H, CH_2); ^{13}C NMR (150 MHz, $CDCl_3$) δ: 166.1, 163.1, 154.8, 148.9, 144.9, 137.7, 132.1, 128.0, 127.8, 125.9, 123.9, 121.8, 121.2, 111.5, 63.0, 51.4, 51.3, 26.7, 24.0; IR (KBr) υ: 3459, 3391, 3061, 3030, 2949, 2837, 1701, 1606, 1512, 1446, 1377, 1285, 1247, 1192, 1154, 1086, 1014, 938, 850, 755cm^{-1}; HRMS (ESI) Calcd. for $C_{24}H_{23}N_3NaO_3S$ ($[M+Na]^+$): 456.1352. Found: 456.1344.

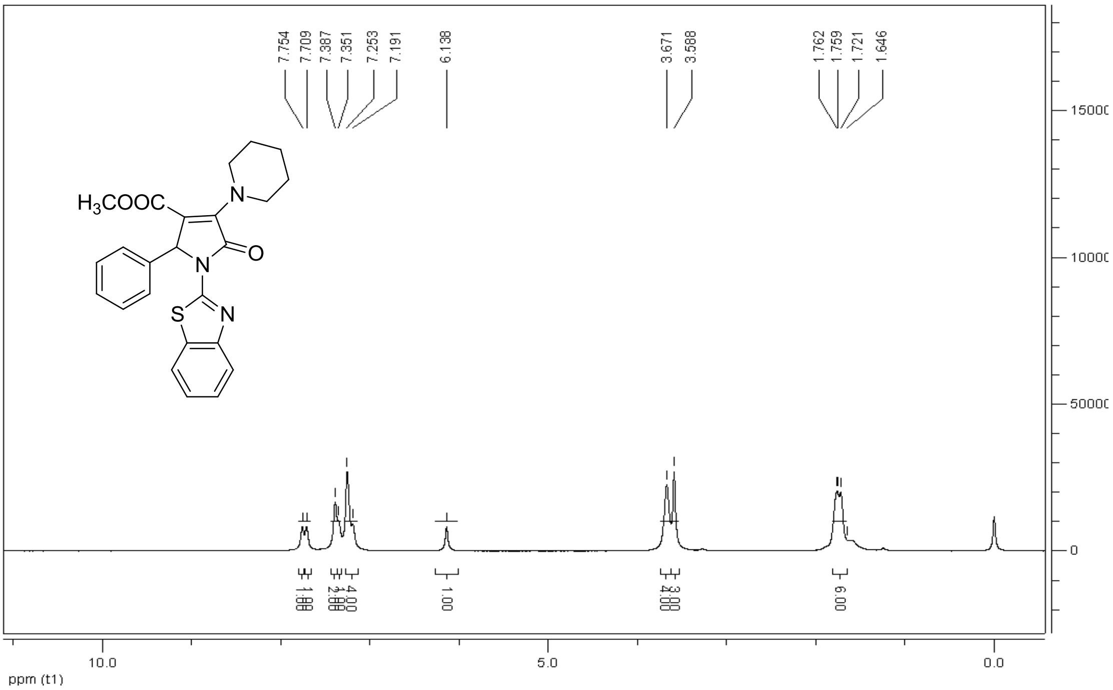

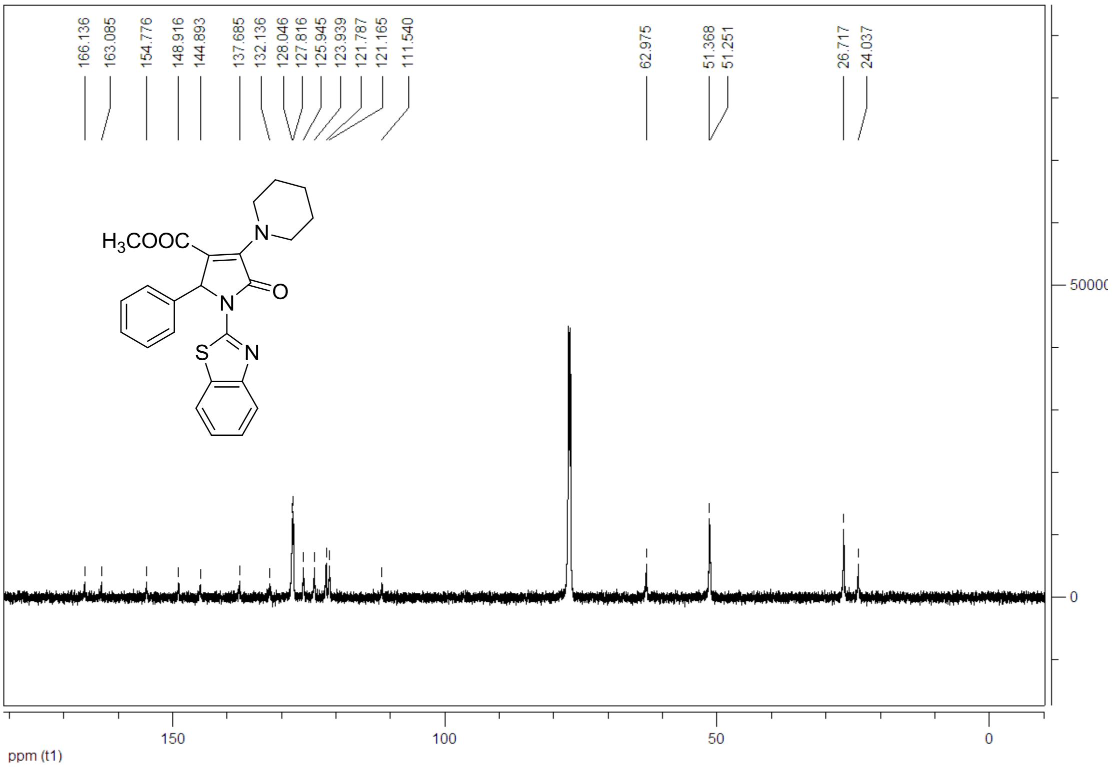

Methyl 1-(benzo[*d*]thiazol-2-yl)-5-oxo-4-(piperidin-1-yl)-2-*m*-tolyl-2,5-dihydro-1*H*-pyrrole-3-carboxylate (1b): white solid, 72%, m.p. 171.1~171.7℃; ^{1}H NMR (600 MHz, DMSO) δ: 7.98 (d, *J* = 7.8Hz, 1H, ArH), 7.65 (d, *J* = 7.8Hz, 1H, ArH), 7.39 (t, *J* = 7.8Hz, 1H, ArH), 7.30 (t, *J* = 7.8Hz, 1H, ArH), 7.16 (d, *J* = 4.8Hz, 2H, ArH), 7.13 (s, 1H, ArH), 7.00 (d, *J* = 3.6Hz, 1H, ArH), 6.00 (s, 1H, CH), 3.63 (d, *J* = 14.4Hz, 2H, CH_2), 3.52 (s, 3H, OCH_3), 3.46 (t, *J* = 6.0Hz, 2H, CH_2), 2.24 (s, 3H, CH_3), 1.66 (d, *J* = 4.2Hz, 6H, CH_2); ^{13}C NMR (150 MHz, DMSO) δ: 165.6, 162.3, 154.5, 148.2, 143.6, 137.6, 137.0, 131.3, 128.5, 127.8, 127.7, 126.2, 124.9, 124.1, 121.7, 121.2, 111.9, 62.4, 51.0, 50.6, 26.1, 23.4, 21.0; IR (KBr) υ: 3454, 3062, 3025, 2942, 2851, 1700, 1607, 1511, 1444, 1377, 1284, 1245, 1191, 1157, 1112, 1084, 1012, 939, 848, 808, 754cm^{-1}; HRMS (ESI) Calcd. for $C_{25}H_{26}N_3O_3S$ ($[M+H]^+$): 448.1689. Found: 448.1684.

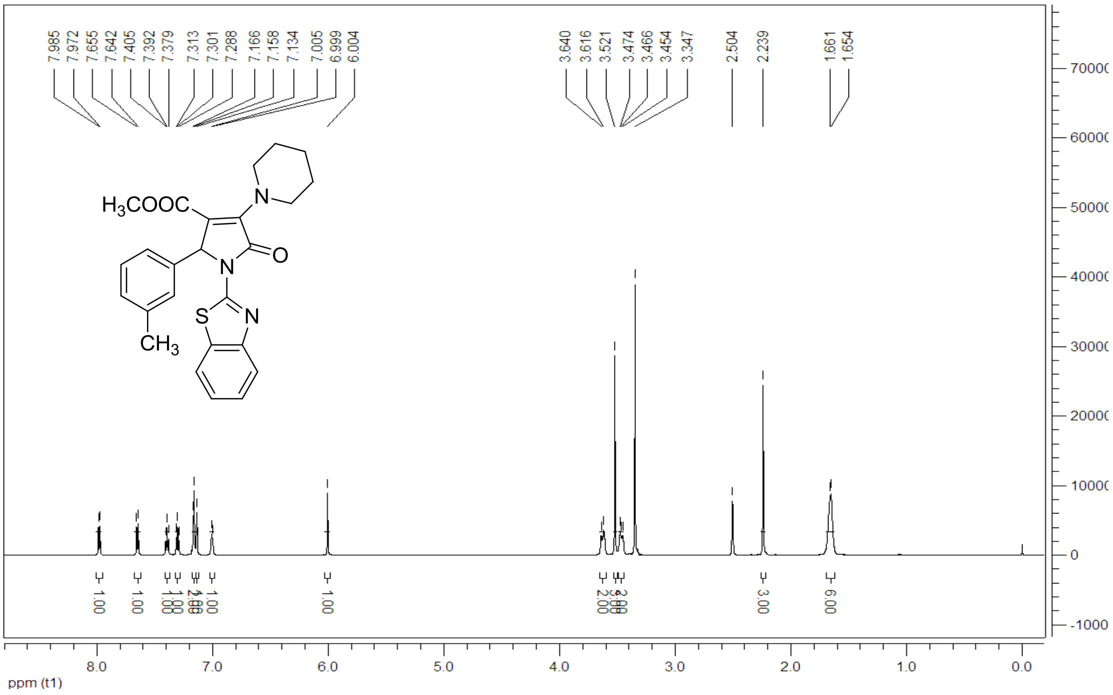

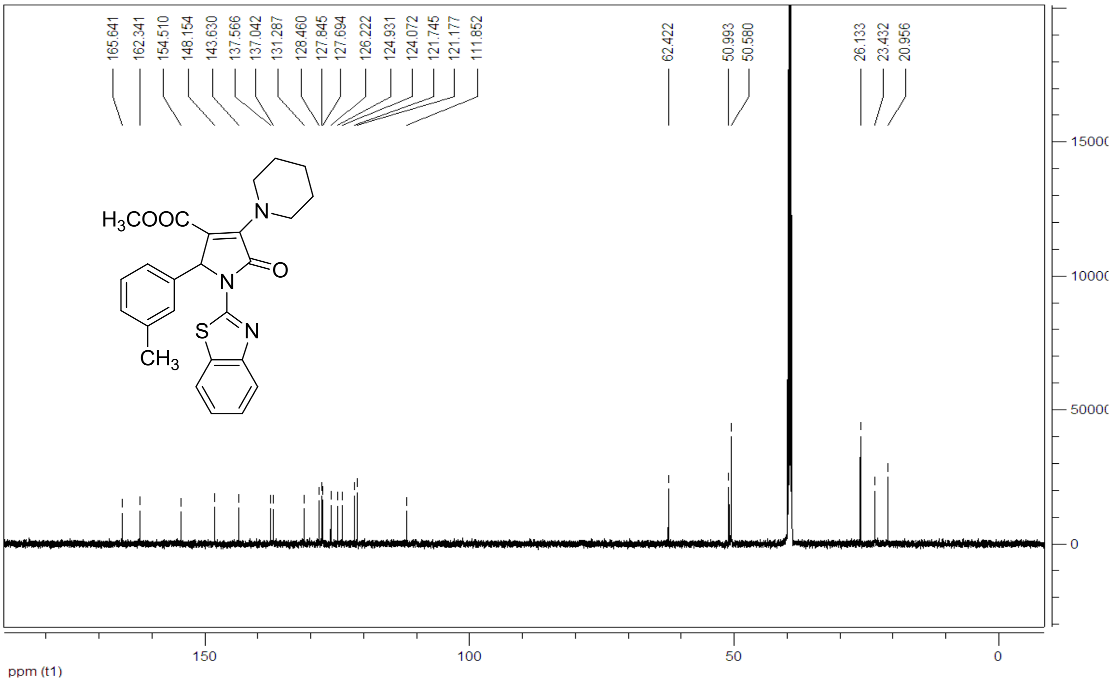

Methyl 1-(benzo[*d*]thiazol-2-yl)-2-(4-isopropylphenyl)-5-oxo-4-(piperidin-1-yl)-2,5-dihydro-1*H*-pyrrole-3-carboxylate (1c): white solid, 55%, m.p. 203.4~203.7℃; ^{1}H NMR (600 MHz, $CDCl_3$) δ: 7.77~7.72 (m, 2H, ArH), 7.35 (t, *J* = 7.8Hz, 1H, ArH), 7.29 (d, *J* = 7.8Hz, 2H, ArH), 7.23 (d, *J* = 7.8Hz, 1H, ArH), 7.09 (d, *J* = 7.8Hz, 2H, ArH), 6.13 (s, 1H, CH), 3.66 (s, 4H, CH_2), 3.59 (s, 3H, OCH_3), 2.84~2.79 (m, 1H, CH), 1.78~1.71 (m, 6H, CH_2), 1.17 (d, *J* = 6.6Hz, 6H, CH_3); ^{13}C NMR (150 MHz, $CDCl_3$) δ: 166.2, 163.1, 154.8, 149.0, 148.2, 144.8, 134.7, 132.1, 127.6, 126.1, 125.9, 123.9, 121.8, 121.1, 111.9, 62.7, 51.3, 51.2, 33.7, 26.7, 24.0, 23.8; IR (KBr) υ: 3687, 3434, 3062, 2953, 1702, 1609, 1512, 1446, 1376, 1283, 1247, 1194, 1155, 1089, 1015, 939, 895, 841, 756cm^{-1}; HRMS (ESI) Calcd. for $C_{27}H_{29}N_3NaO_3S$ ($[M+Na]^+$): 498.1822. Found: 498.1813.

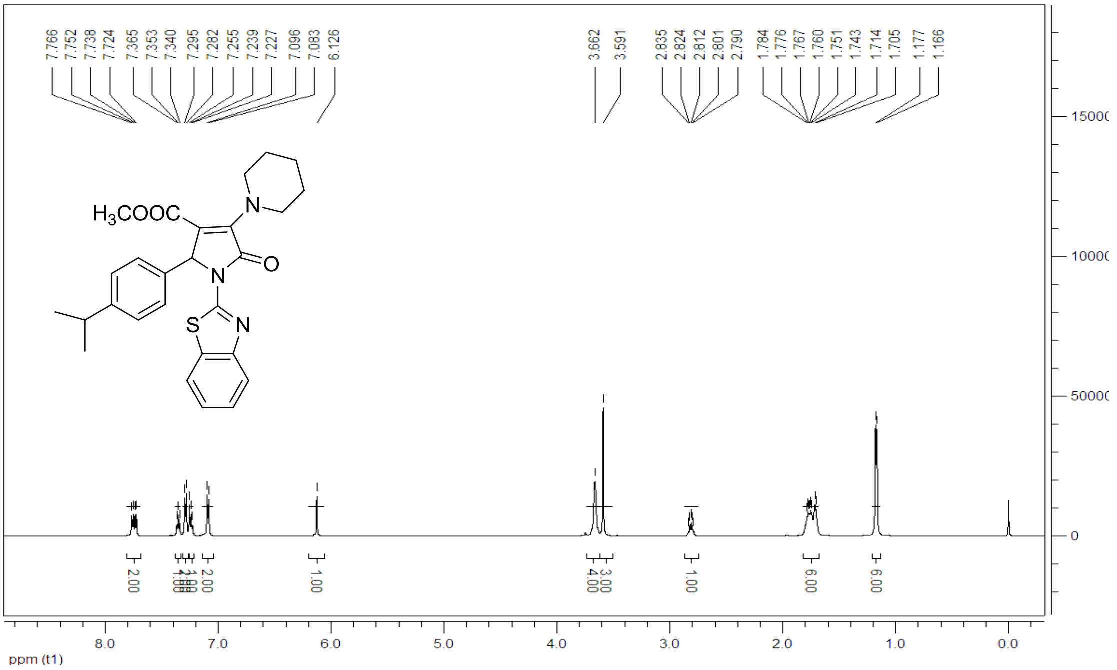

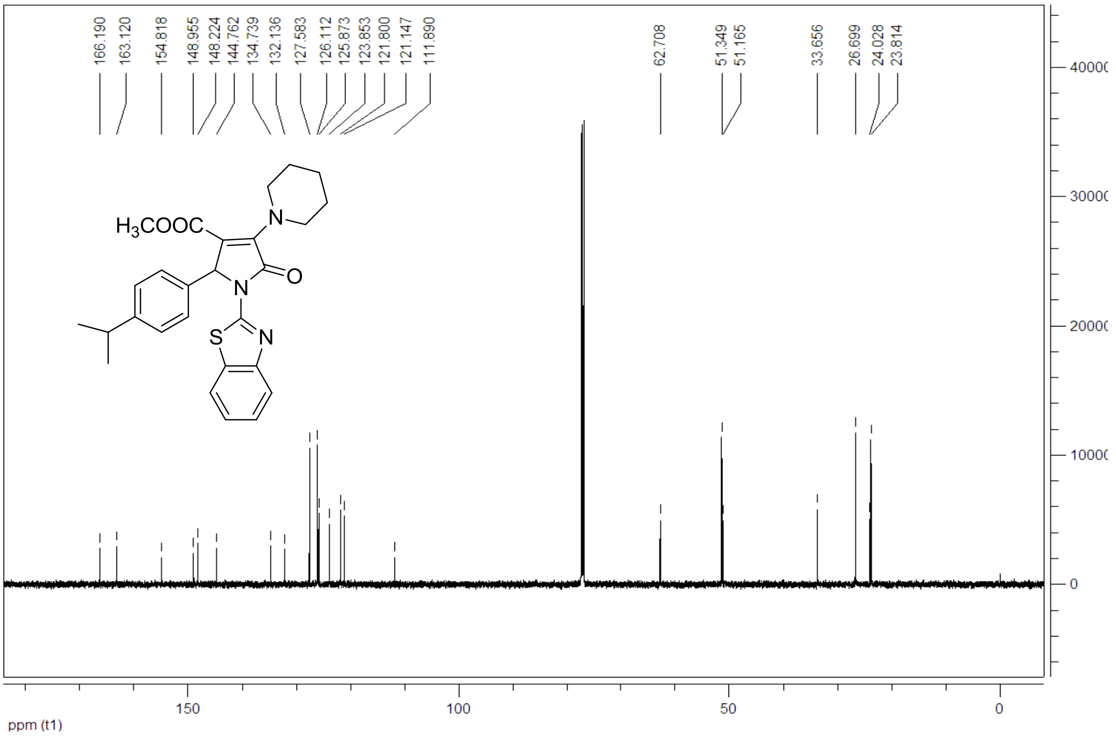

Methyl 1-(benzo[*d*]thiazol-2-yl)-2-(4-chlorophenyl)-5-oxo-4-(piperidin-1-yl)-2,5-dihydro-1*H*-pyrrole-3-carboxylate (1d): white solid, 53%, m.p. 208.4~208.7℃; ^{1}H NMR (600 MHz, DMSO) δ: 7.99 (s, 1H, ArH), 7.66 ~7.58(m, 1H, ArH), 7.40 ~7.20(m, 6H, ArH), 6.04 (s, 1H, CH), 3.61~3.53 (m, 7H, OCH_3, CH_2), 1.67 (s, 6H, CH_2); ^{13}C NMR (150 MHz, DMSO) δ: 165.5, 162.2, 154.5, 148.1, 144.0, 137.0, 132.1, 131.3, 129.6, 128.0, 126.3, 124.1, 121.8, 121.2, 110.9, 61.8, 51.0, 50.6, 26.1, 23.4; IR (KBr) υ: 3453, 2940, 2851, 1702, 1610, 1512, 1449, 1377, 1287, 1186, 1092, 1016, 938, 841, 758cm^{-1}; HRMS (ESI) Calcd. for $C_{24}H_{23}ClN_3O_3S$ ($[M+H]^+$): 468.1143. Found: 468.1131.

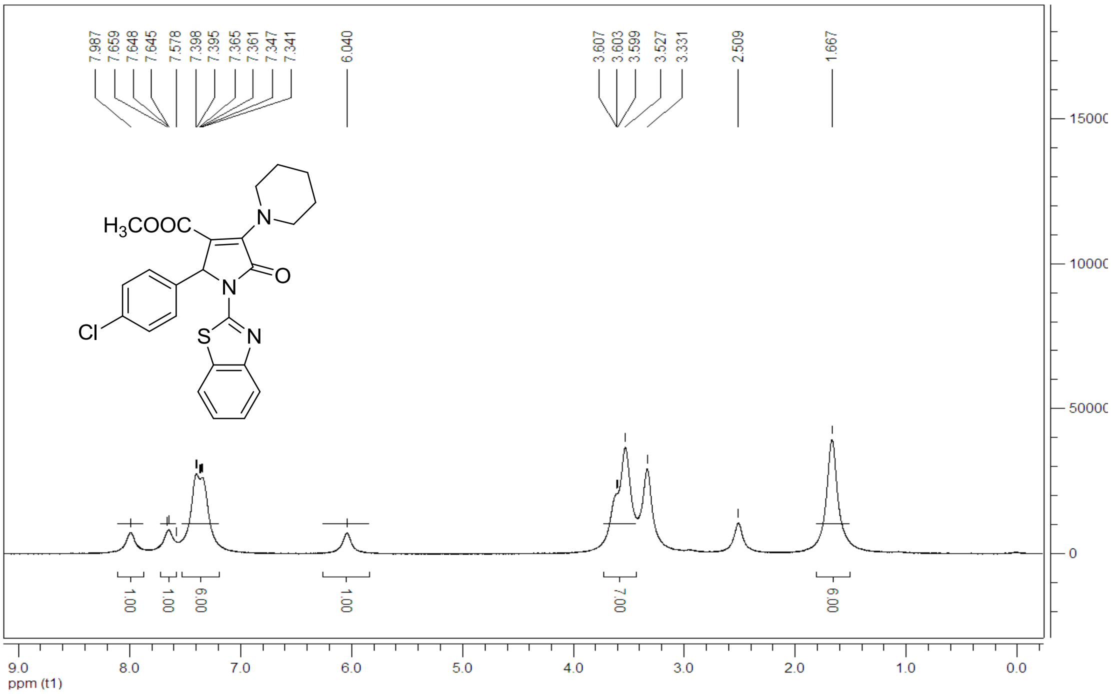

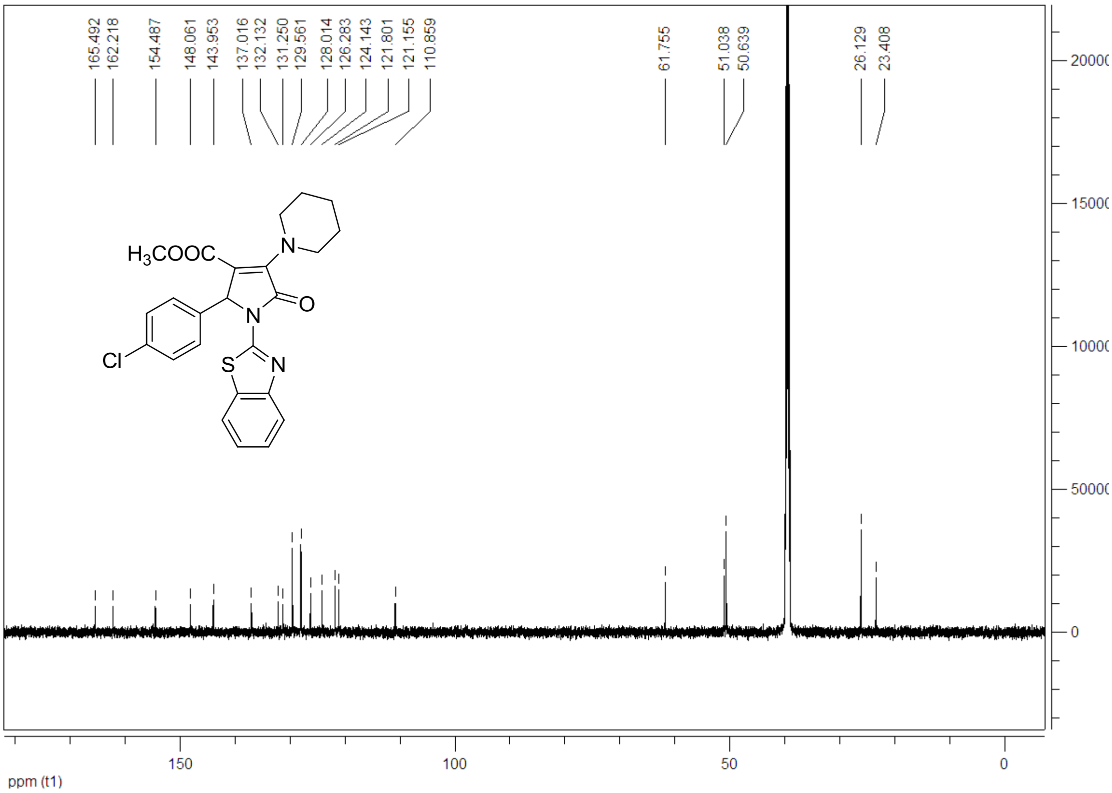

Methyl 1-(benzo[*d*]thiazol-2-yl)-2-(3-chlorophenyl)-5-oxo-4-(piperidin-1-yl)-2,5-dihydro-1*H*-pyrrole-3-carboxylate (**1e**): white solid, 67%, m.p. 181.1~181.6℃; ^{1}H NMR (600 MHz, $CDCl_3$) δ: 7.77 (s, 1H, ArH), 7.73 (s, 1H, ArH), 7.38 (s, 3H, ArH), 7.17 (s, 3H, ArH), 6.09 (s, 1H, CH), 3.69 (s, 4H, CH_2), 3.61 (s, 3H, OCH_3), 1.77~1.65 (m, 6H, CH_2); ^{13}C NMR (150 MHz, $CDCl_3$) δ: 165.9, 162.8, 154.6, 148.8, 145.0, 140.1, 133.8, 132.1, 129.3, 128.1, 128.0, 126.1, 124.1, 121.8, 121.2, 110.4, 62.4, 51.4, 51.3, 26.7, 24.0; IR (KBr) υ: 3680, 3372, 3061, 3027, 2942, 2849, 1903, 1702, 1606, 1510, 1441, 1372, 1283, 1245, 1188, 1080, 1007, 936, 903, 852, 803, 754cm^{-1}; HRMS (ESI) Calcd. for $C_{24}H_{23}ClN_3O_3S$ ($[M+H]^+$): 468.1143. Found: 468.1135.

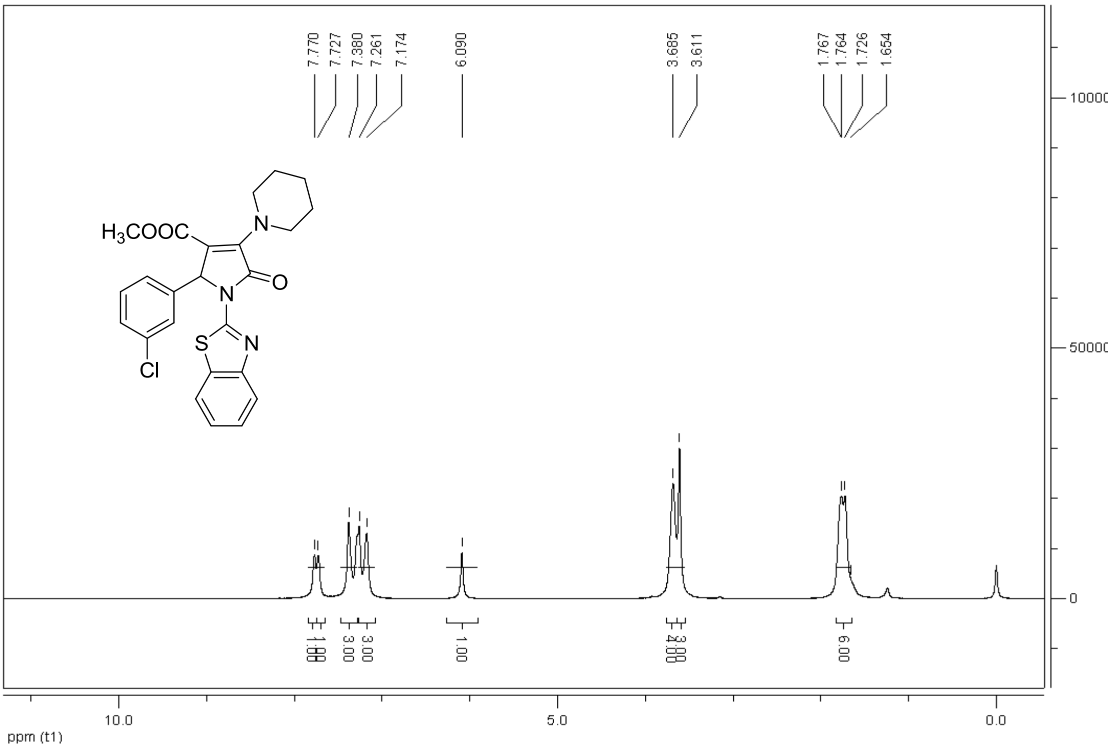

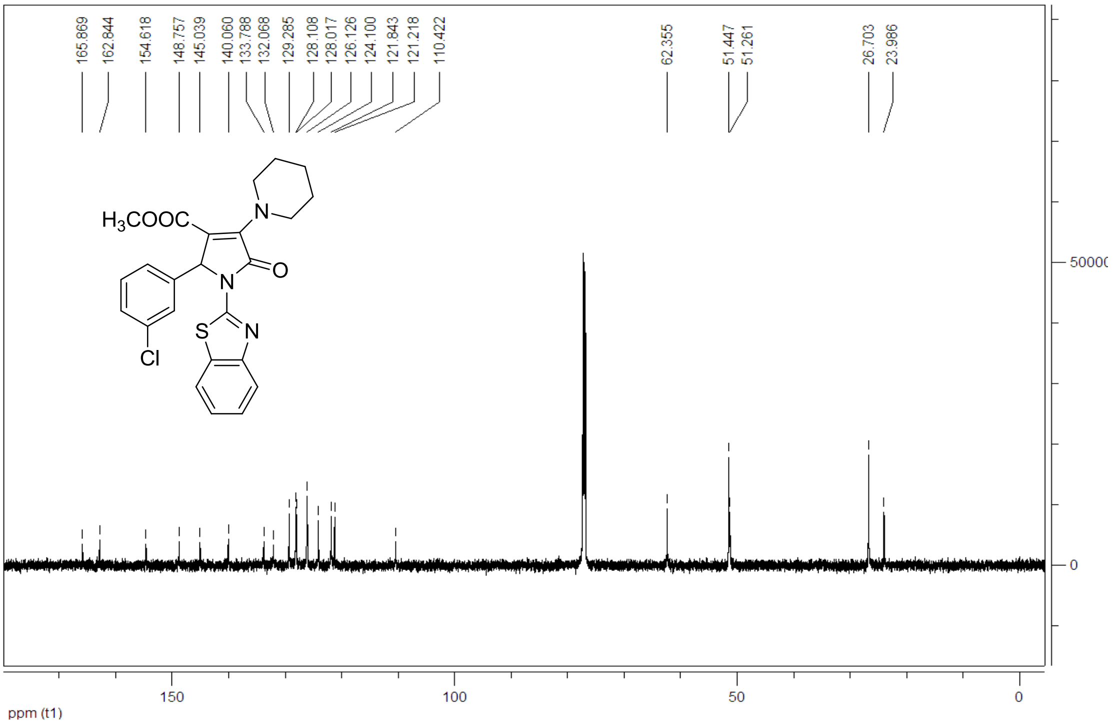

Methyl 1-(benzo[*d*]thiazol-2-yl)-2-(3-nitrophenyl)-5-oxo-4-(piperidin-1-yl)-2,5-dihydro-1*H*-pyrrole-3-carboxylate (1f): light yellow solid, 70%, m.p. 205.8~206.3℃; ^{1}H NMR (600 MHz, DMSO) δ: 8.30 (s, 1H, ArH), 8.09 (d, *J* = 4.8Hz, 1H, ArH), 7.99 (d, *J* = 5.4Hz, 1H, ArH), 7.86 (s, 1H, ArH), 7.60 (s, 2H, ArH), 7.39 (s,1H, ArH), 7.30 (s,1H, ArH), 6.20 (s, 1H, CH), 3.65 (s, 2H, CH_2), 3.55 (s, 2H, CH_2), 3.51 (s, 3H, OCH_3), 1.68 (s, 6H, CH_2); ^{13}C NMR (150 MHz, DMSO) δ: 165.4, 162.1, 154.5, 147.9, 147.4, 144.4, 140.6, 134.2, 131.2, 129.6, 126.3, 124.2, 123.0, 122.8, 121.8, 121.1, 109.5, 61.7, 51.0, 50.7, 26.2, 23.4; IR (KBr) υ: 3482, 3388, 3040, 2988, 2931, 2855, 1963, 1903, 1795, 1702, 1605, 1520, 1446, 1375, 1350, 1304, 1187, 1094, 1016, 941, 852, 816, 757cm^{-1}; HRMS (ESI) Calcd. for $C_{24}H_{22}N_4NaO_5S$ ($[M+Na]^+$): 501.1203. Found: 501.1199.

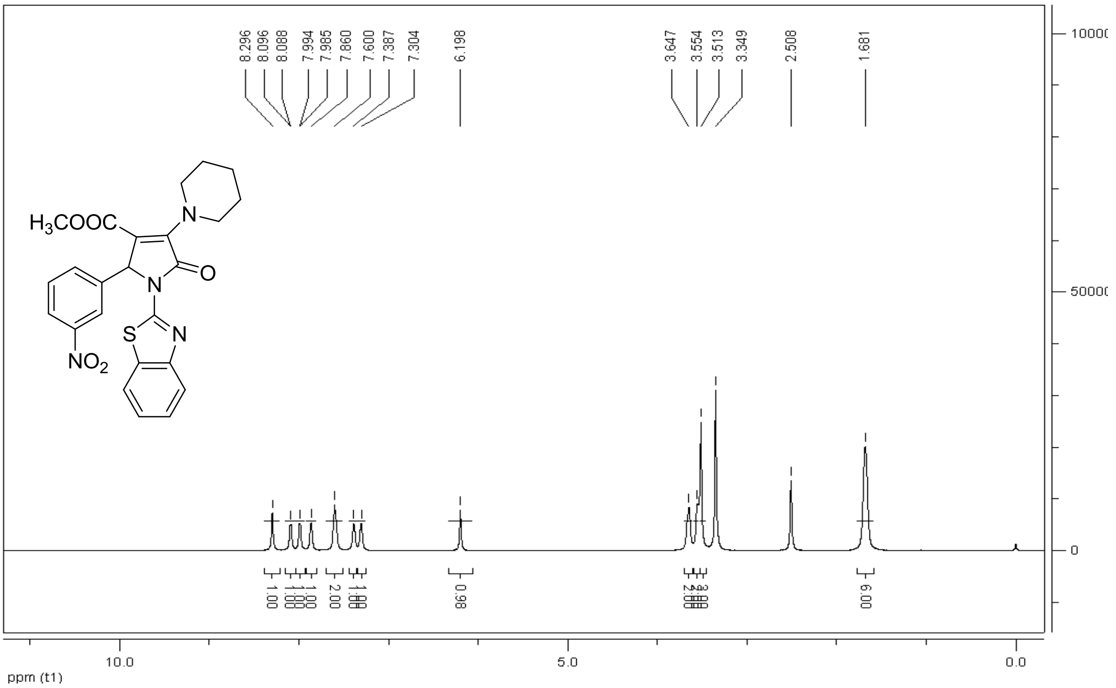

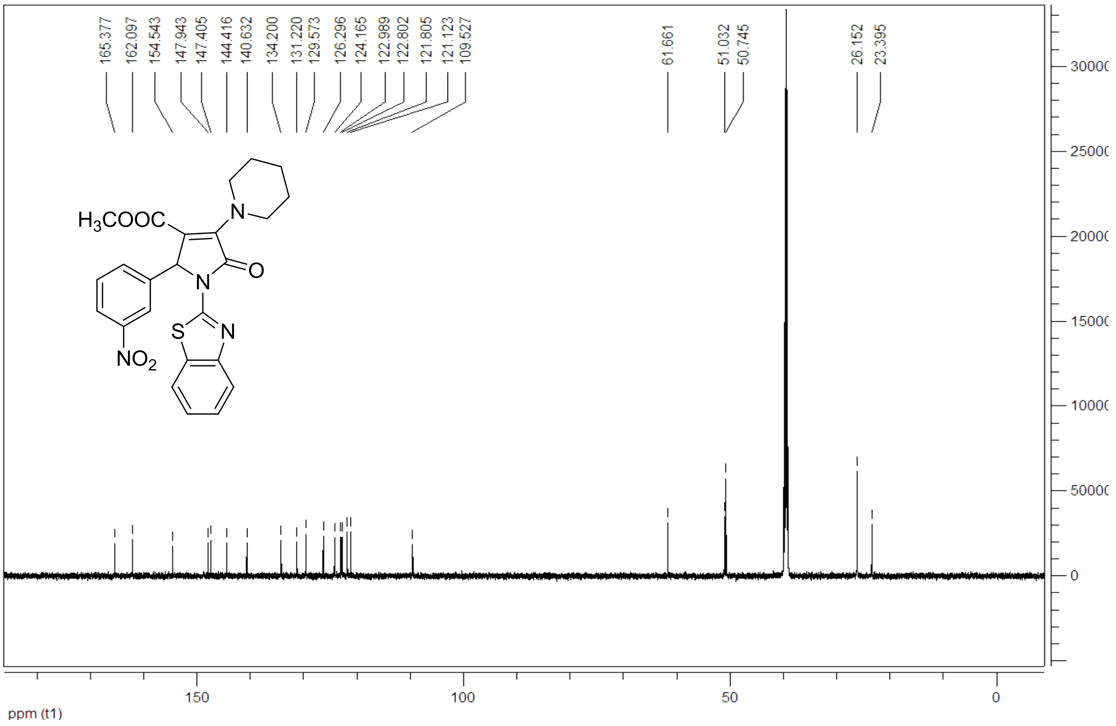

Ethyl 1-(benzo[d]thiazol-2-yl)-5-oxo-4-(piperidin-1-yl)-2-*p*-tolyl-2,5-dihydro-1H-pyrrole-3-carboxylate (1g): white solid, 63%, m.p. 192.7~192.9℃; ^{1}H NMR (600 MHz, $CDCl_3$) δ: 7.74 (d, *J* = 13.8Hz, 2H, ArH), 7.35 (s, 4H, ArH), 7.05 (s, 2H, ArH), 6.11 (s, 1H, CH), 4.06 (s, 1H, CH), 4.01 (s, 1H, CH), 3.69 (s, 2H, CH_2), 3.62 (s, 2H, CH_2), 2.25 (s, 3H, CH_3), 1.76 (s, 4H, CH_2), 1.71 (s, 2H, CH_2), 1.12 (s, 3H, CH_3); ^{13}C NMR (150 MHz, $CDCl_3$) δ: 166.3, 162.7, 154.9, 148.9, 144.7, 137.5, 134.6, 132.1, 128.7, 127.9, 125.9, 123.9, 121.8, 121.2, 112.2, 62.8, 60.2, 51.3, 26.7, 24.1, 21.2, 14.0; IR (KBr) υ: 3700, 3339, 2945, 2837, 1901, 1698, 1607, 1512, 1444, 1375, 1284, 1245, 1196, 1157, 1086, 1015, 961, 831, 755cm^{-1}; HRMS (ESI) Calcd. for $C_{26}H_{27}N_3NaO_3S$ ($[M+Na]^+$): 484.1665. Found: 484.1658.

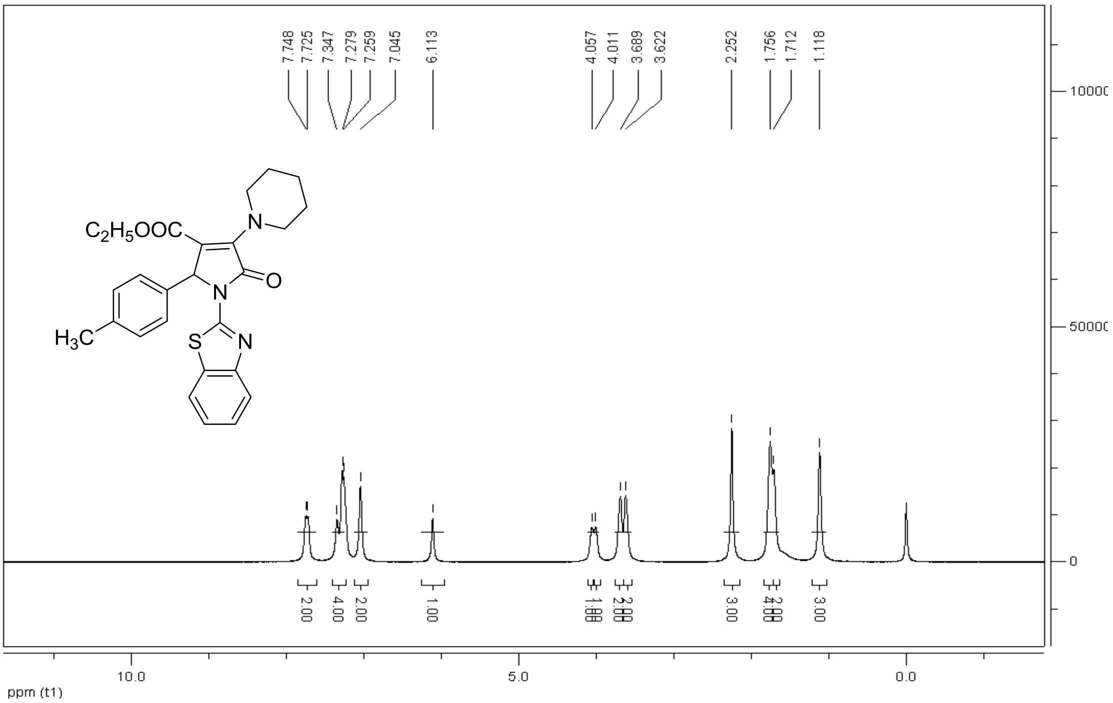

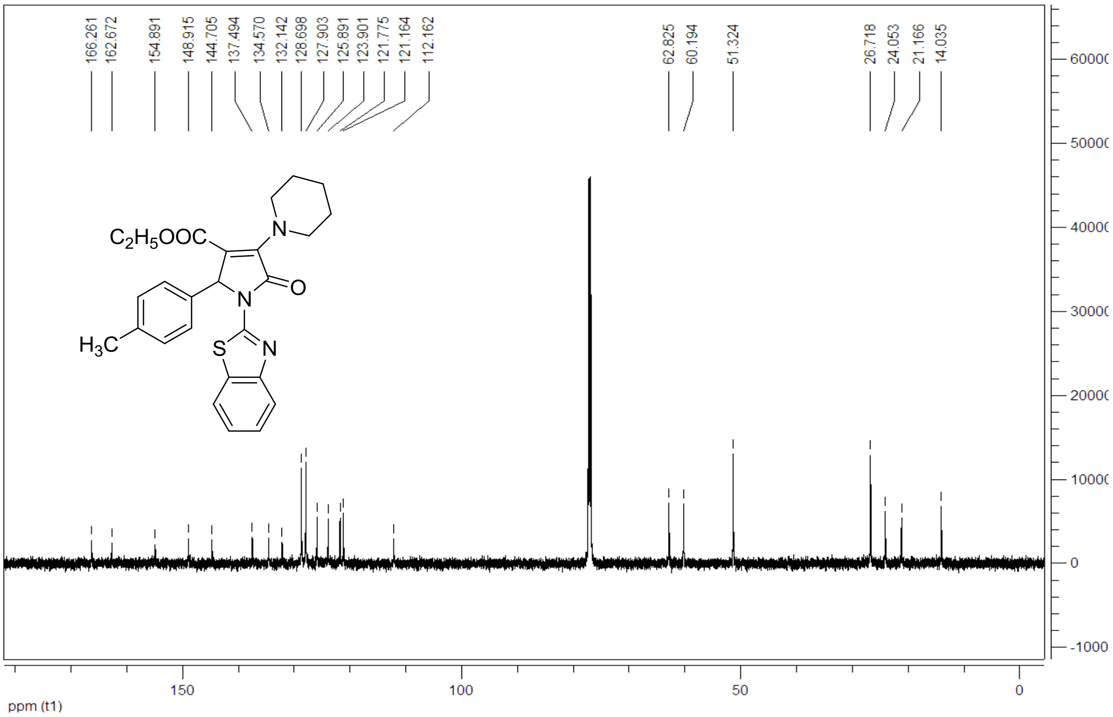

Methyl 1-(benzo[*d*]thiazol-2-yl)-5-oxo-2-phenyl-4-(pyrrolidin-1-yl)-2,5-dihydro-1*H*-pyrrole-3-carboxylate (1h): white solid, 66%, m.p. 250.3~250.7; ^{1}H NMR (600 MHz, $CDCl_3$) δ: 7.76~7.70 (m, 2H, ArH), 7.40 (s, 2H, ArH), 7.35 (s, 1H, ArH), 7.18 (t, 4H, ArH), 6.17 (s, 1H, CH), 3.93 (s, 2H, CH_2), 3.85 (s, 2H, CH_2), 3.53 (s, 3H, OCH_3), 1.96 (t, J = 7.2Hz, 4H, CH_2); ^{13}C NMR (150 MHz, $CDCl_3$) δ: 165.6, 163.5, 154.8, 148.9, 141.2, 138.3, 132.2, 127.9, 127.8, 127.7, 125.9, 123.9, 121.8, 121.1, 106.9, 63.5, 51.7, 50.9, 25.6; IR (KBr) υ: 3396, 3062, 3032, 2985, 2947, 2878, 1704, 1610, 1513, 1445, 1374, 1340, 1316, 1279, 1244, 1201, 1176, 1157, 1117, 1093, 1034, 955, 897, 873, 829, 808, 759cm^{-1}; HRMS (ESI) Calcd. for $C_{23}H_{22}N_3O_3S$ ([M+H]$^+$): 420.1376. Found: 420.1364.

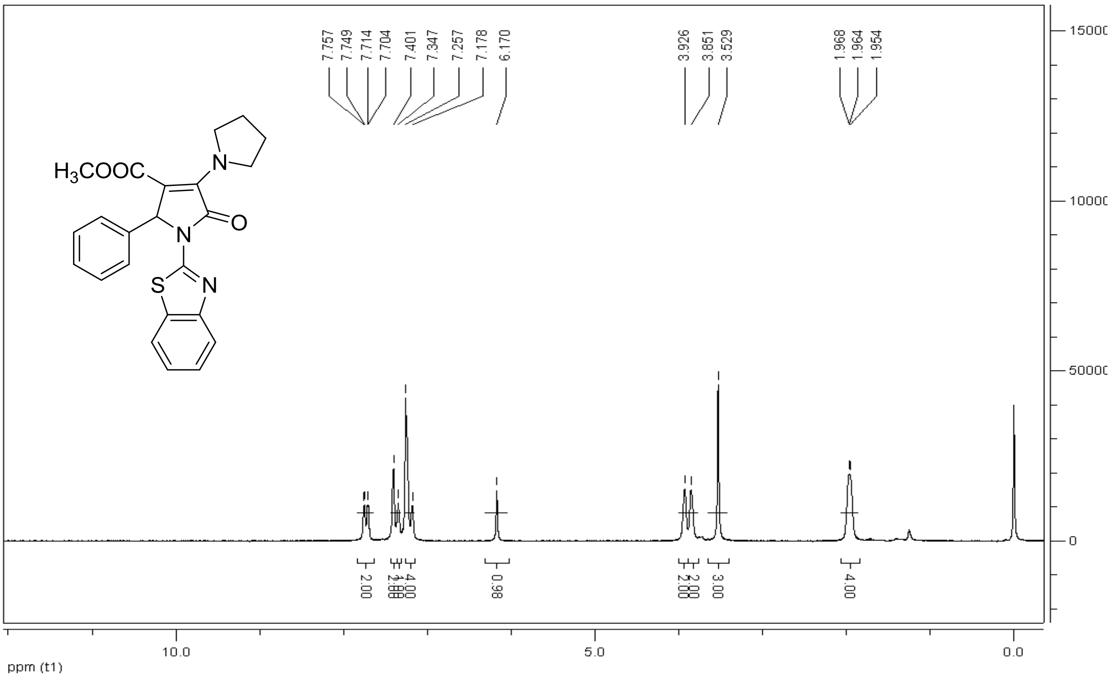

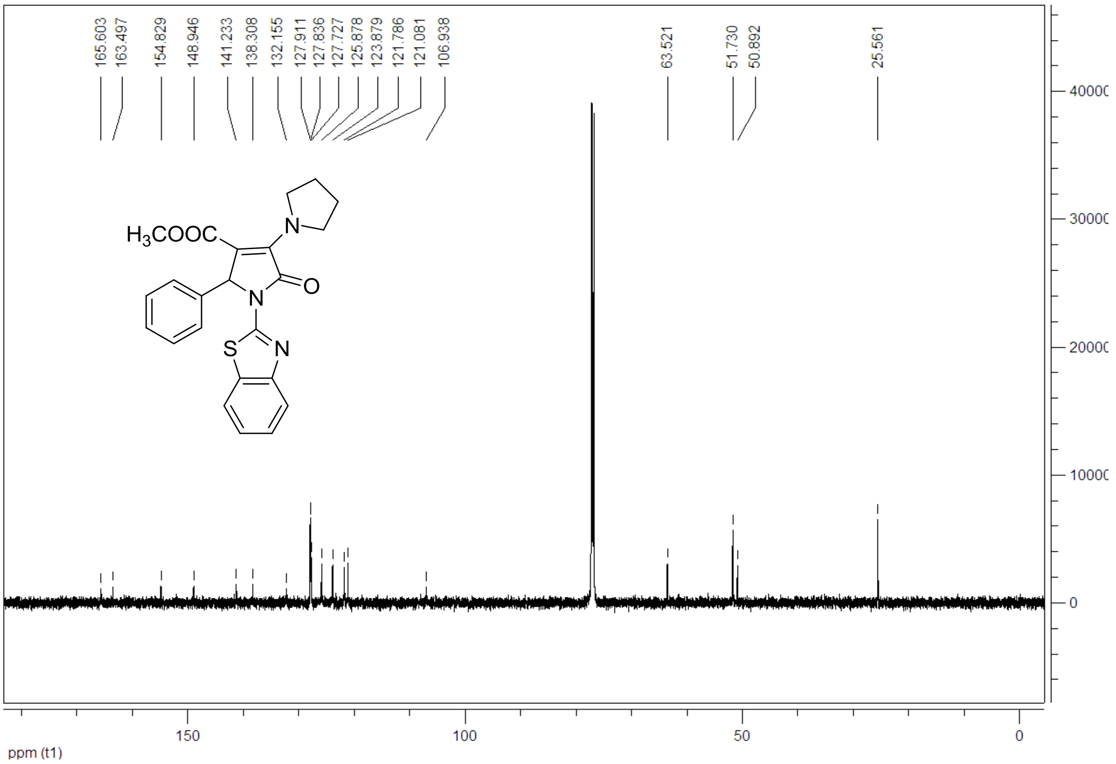

Methyl 1-(benzo[*d*]thiazol-2-yl)-2-(4-methoxyphenyl)-5-oxo-4-(pyrrolidin-1-yl)-2,5-dihydro-1*H*-pyrrole-3-carboxylate (1i): white solid, 55%, m.p. 220.9~221.3℃; ^{1}H NMR (600 MHz, DMSO) δ: 7.98 (s, 1H, ArH), 7.67 (s, 1H, ArH), 7.40~7.30 (m, 4H, ArH), 6.82 (s, 2H, ArH), 6.03 (s, 1H, CH), 3.80~3.69 (m, 7H, CH_2, OCH_3), 3.47 (s, 3H, OCH_3), 1.93~1.87 (m, 4H, CH_2); ^{13}C NMR (150 MHz, DMSO) δ: 165.5, 163.2, 159.1, 148.7, 140.8, 131.8, 130.5, 129.3, 126.7, 124.5, 122.2, 121.7, 113.8, 107.2, 63.0, 55.4, 51.8, 51.2, 25.5; IR (KBr) υ: 3693, 3434, 3058, 2986, 2944, 2874, 1702, 1609, 1512, 1449, 1372, 1337, 1283, 1246, 1210, 1173, 1116, 1029, 954, 899, 837, 759cm^{-1}; HRMS (ESI) Calcd. for $C_{24}H_{23}N_3NaO_4S$ ($[M+Na]^+$): 472.1301. Found: 472.1291.

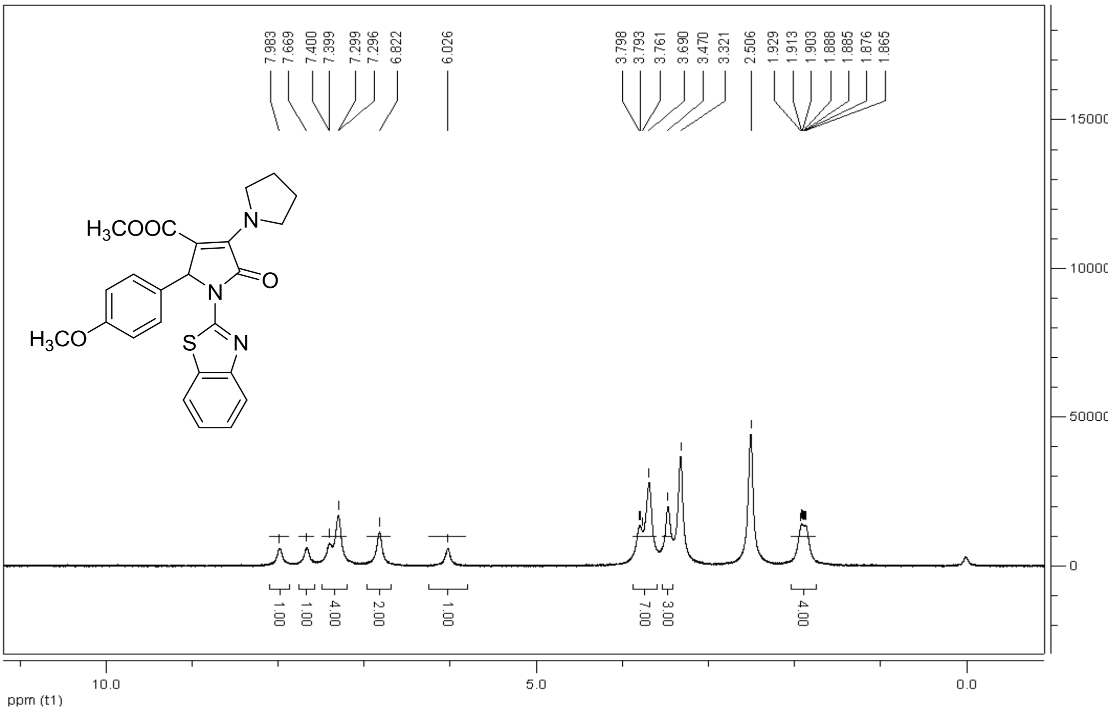

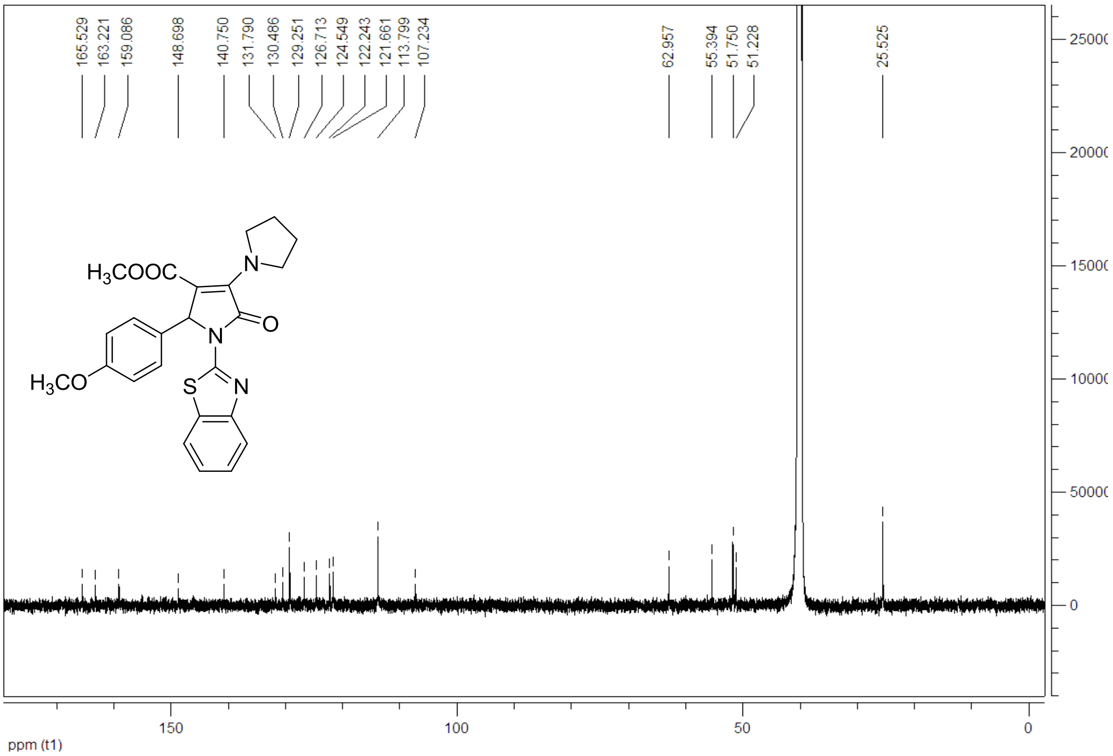

Methyl 1-(benzo[*d*]thiazol-2-yl)-2-(4-chlorophenyl)-5-oxo-4-(pyrrolidin-1-yl)-2,5-dihydro-1*H*-pyrrole-3-carboxylate (1j): white solid, 58%, m.p. 225.6~226.5℃; ^{1}H NMR (600 MHz, DMSO) δ: 7.99 (d, *J* = 8.4Hz, 1H, ArH), 7.64 (d, *J* = 7.8Hz, 1H, ArH), 7.43~7.38 (m, 3H, ArH), 7.33~7.29 (m, 3H, ArH), 6.06 (s, 1H, CH), 3.79~3.73 (m, 4H, CH_2), 3.47 (s, 3H, OCH_3), 1.90~1.86 (m, 4H, CH_2); ^{13}C NMR (150 MHz, DMSO) δ: 164.9, 162.5, 154.5, 148.1, 140.7, 137.7, 132.0, 131.2, 129.6, 127.9, 126.3, 124.1, 121.8, 121.1, 105.8, 62.2, 51.4, 50.7, 25.0; IR (KBr) υ: 3452, 3055, 2948, 2875, 1701, 1610, 1511, 1446, 1372, 1330, 1282, 1247, 1205, 1092, 1018, 951, 898, 833, 759cm^{-1}; HRMS (ESI) Calcd. for $C_{23}H_{21}ClN_3O_3S$ ($[M+H]^+$): 454.0987. Found: 454.0978.

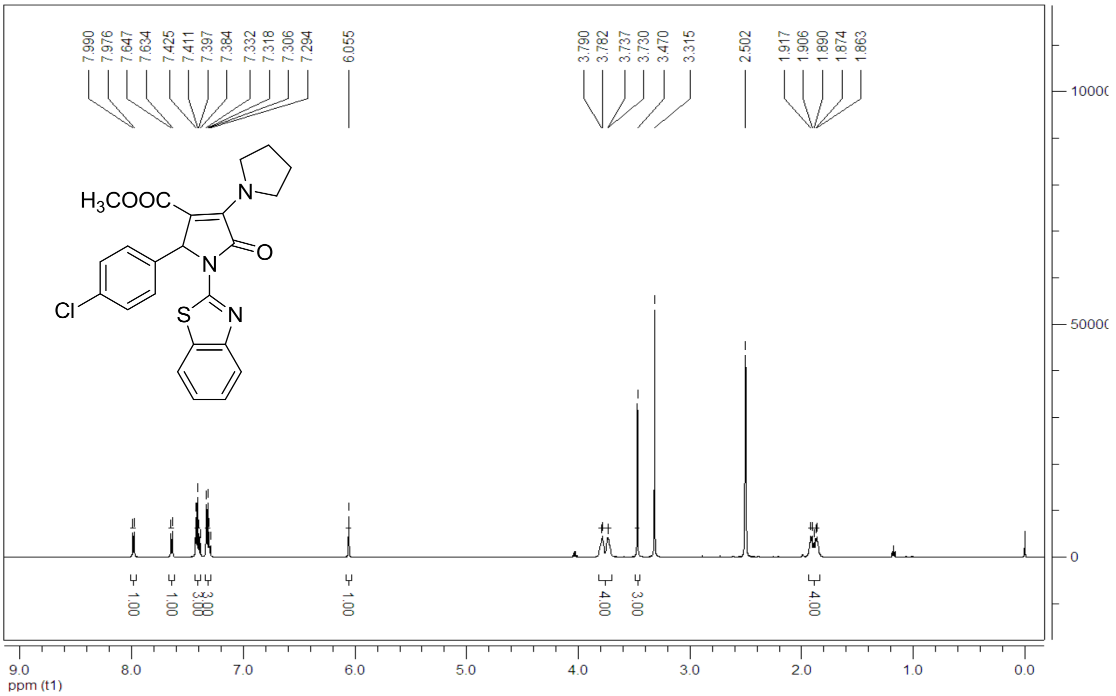

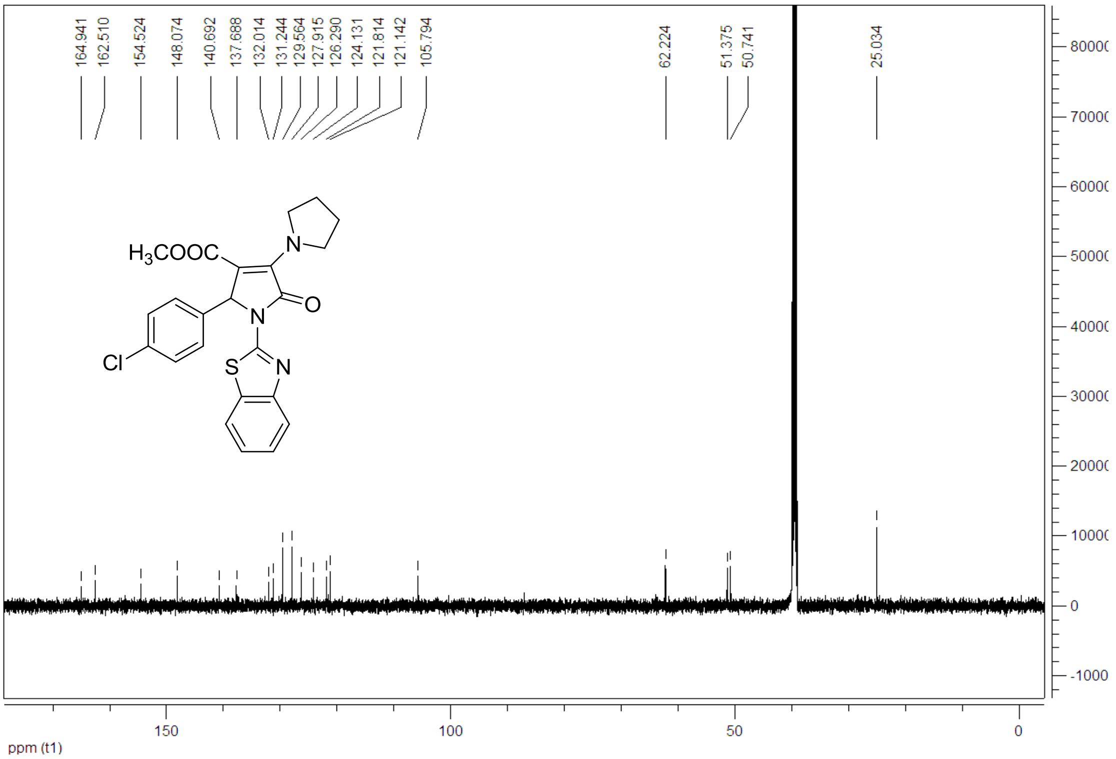

Methyl 1-(benzo[*d*]thiazol-2-yl)-2-(3-chlorophenyl)-5-oxo-4-(pyrrolidin-1-yl)-2,5-dihydro-1*H*-pyrrole-3-carboxylate (1k): white solid, 63%, m.p. 173.6~174.1℃; ^{1}H NMR (600 MHz, $CDCl_3$) δ: 7.77 (d, *J* = 7.2Hz, 1H, ArH), 7.72 (d, *J* = 7.8Hz, 1H, ArH), 7.40~7.36 (m, 2H, ArH), 7.31 (d, *J* = 6.0Hz, 1H, ArH), 7.26 (s, 1H, ArH), 7.17 (d, *J* = 14.4Hz, 2H, ArH), 6.13 (s, 1H, CH), 3.93 (s, 2H, CH_2), 3.87 (s, 2H, CH_2), 3.56 (s, 3H, OCH_3), 1.97 (t, *J* = 6.0Hz, 4H, CH_2); ^{13}C NMR (150 MHz, $CDCl_3$) δ: 165.4, 163.3, 154.7, 148.8, 141.4, 140.6, 133.7, 132.1, 129.2, 128.0, 126.2, 126.0, 124.1, 121.8, 121.2, 105.8, 62.9, 51.9, 51.0, 25.6; IR (KBr) υ: 3450, 3057, 2981, 2947, 2873, 1704, 1608, 1573, 1514, 1444, 1369, 1316, 1278, 1245, 1202, 1117, 1094, 1031, 953, 881, 807, 779, 759cm^{-1}; HRMS (ESI) Calcd. for $C_{23}H_{21}ClN_3O_3S$ ($[M+H]^+$): 454.0960. Found: 454.0969.

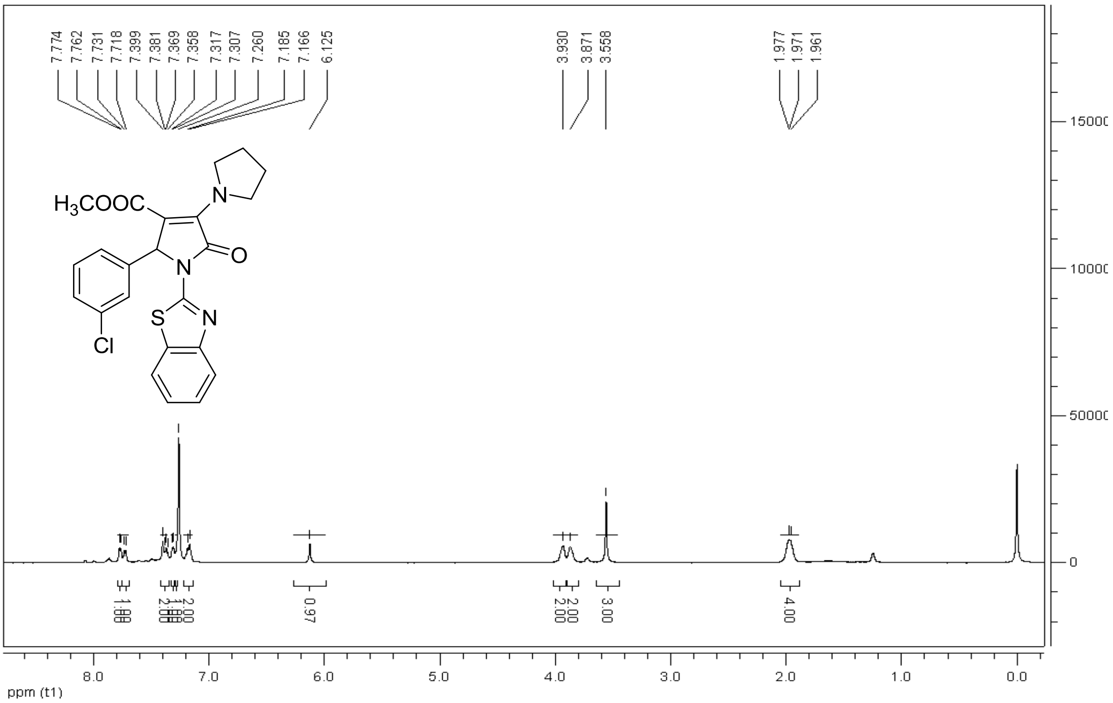

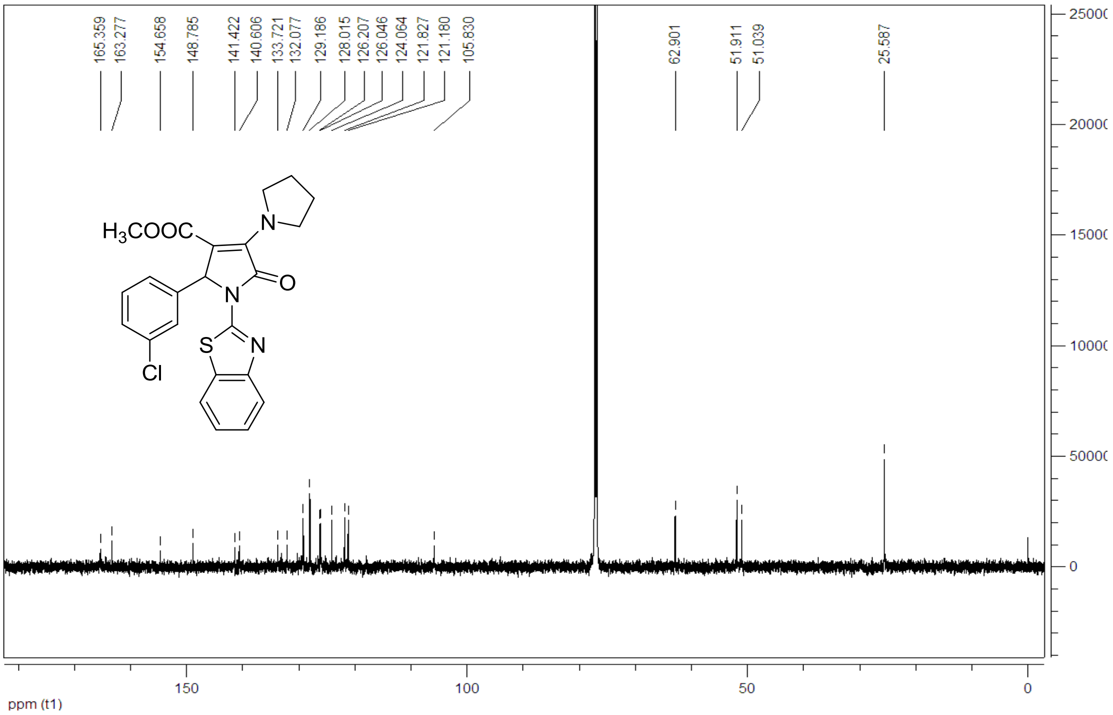

Methyl 1-(benzo[*d*]thiazol-2-yl)-2-(3-nitrophenyl)-5-oxo-4-(pyrrolidin-1-yl)-2,5-dihydro-1*H*-pyrrole-3-carboxylate (1l): light yellow solid, 68%, m.p. 205.1~205.5℃; ^{1}H NMR (600 MHz, $CDCl_3$) δ: 8.35 (s, 1H, ArH), 8.07 (d, *J* = 7.8Hz, 1H, ArH), 7.75 (t, *J* = 7.2Hz, 2H, ArH), 7.69 (d, *J* = 7.8Hz, 1H, ArH), 7.43 (t, *J* = 7.8Hz, 1H, ArH), 7.36 (t, *J* = 7.2Hz, 1H, ArH), 7.26 (s, 1H, ArH), 6.23 (s, 1H, CH), 3.93 (s, 4H, CH_2), 3.55 (s, 3H, OCH_3), 1.99 (d, *J* = 6.0Hz, 4H, CH_2); ^{13}C NMR (150 MHz, $CDCl_3$) δ: 165.1, 163.0, 154.6, 148.6, 148.1, 148.0, 141.8, 141.2, 133.9, 132.0, 131.9, 128.9, 126.2, 124.2, 123.4, 122.9, 121.8, 121.2, 104.8, 62.6, 52.1, 51.1, 25.6; IR (KBr) υ: 3456, 3093, 2980, 2949, 2876, 1704, 1611, 1526, 1441, 1376, 1346, 1294, 1278, 1242, 1226, 1199, 1176, 1096, 1040, 1015, 959, 922, 876, 854, 820, 802, 760cm^{-1}; HRMS (ESI) Calcd. for $C_{23}H_{21}N_4O_5S$ ($[M+H]^+$): 465.1214. Found: 465.1210.

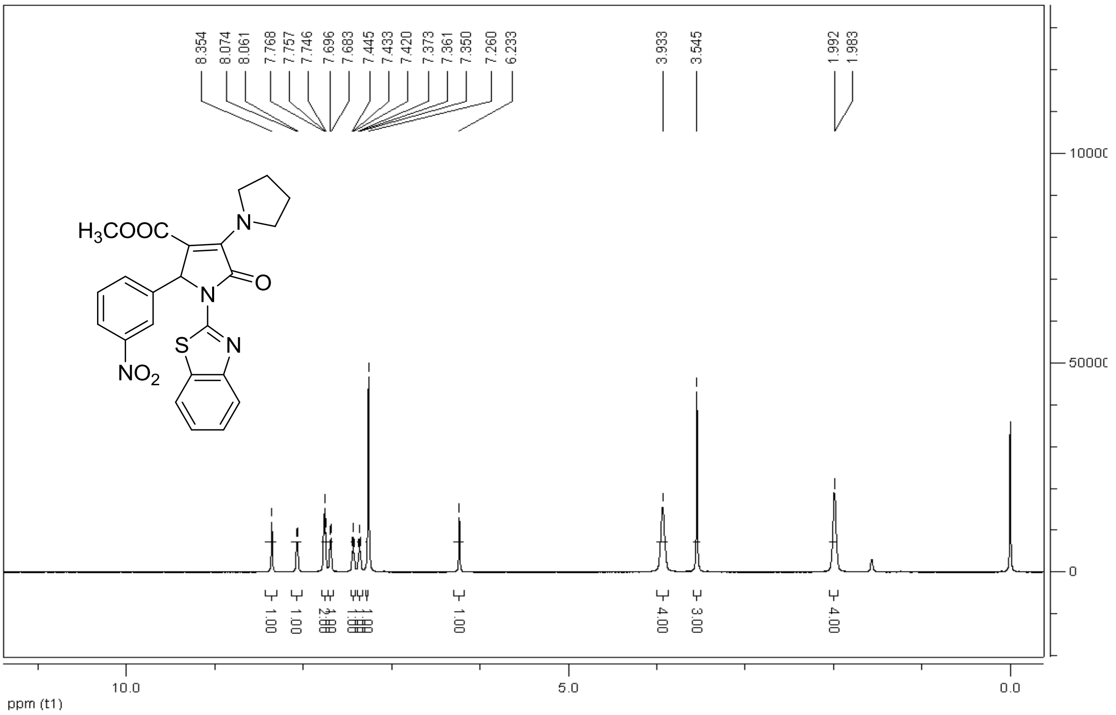

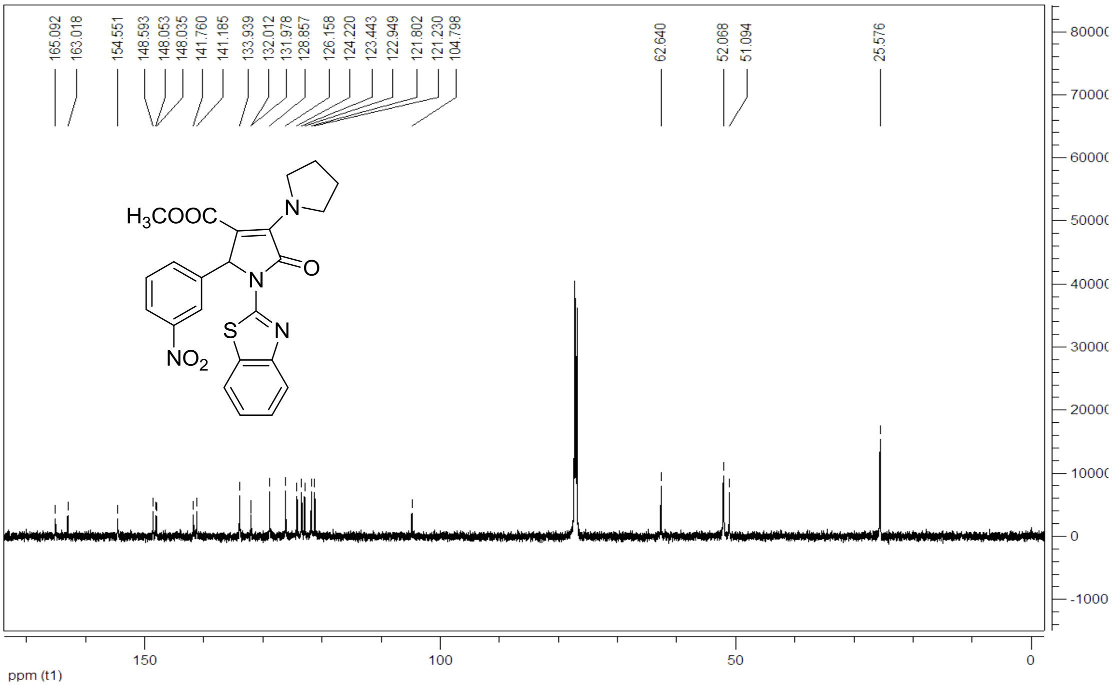

Methyl 1-(benzo[*d*]thiazol-2-yl)-2-(4-nitrophenyl)-5-oxo-4-(pyrrolidin-1-yl)-2,5-dihydro-1*H*-pyrrole-3-carboxylate (1m): white solid, 52%, m.p. 210.7~211.0℃; ^{1}H NMR (600 MHz, $CDCl_3$) δ: 8.13 (d, *J* = 8.4Hz, 2H, ArH), 7.77 (d, *J* = 7.8Hz, 1H, ArH), 7.68 (d, *J* = 8.4Hz, 1H, ArH), 7.60 (d, *J* = 7.8Hz, 2H, ArH), 7.37 (t, *J* = 7.8Hz,, 1H, ArH), 7.28 (s, 1H, ArH), 6.23 (s, 1H, CH), 3.92 (s, 4H, CH_2), 3.55 (s, 3H, OCH_3), 1.99 (d, *J* = 6.0Hz, 4H, CH_2); ^{13}C NMR (150 MHz, $CDCl_3$) δ: 165.2, 163.0, 154.5, 148.5, 147.4, 146.4, 141.7, 131.9, 128.8, 126.2, 124.3, 123.3, 121.7, 121.3, 104.9, 62.6, 52.1, 51.1, 25.6; IR (KBr) υ: 3688, 3435, 3070, 3007, 2956, 1701, 1604, 1516, 1446, 1373, 1345, 1287, 1247, 1194, 1088, 954, 898, 856, 821, 759cm^{-1}; HRMS (ESI) Calcd. for $C_{23}H_{20}N_4NaO_5S$ ($[M+Na]^+$): 487.1047. Found: 487.1040.

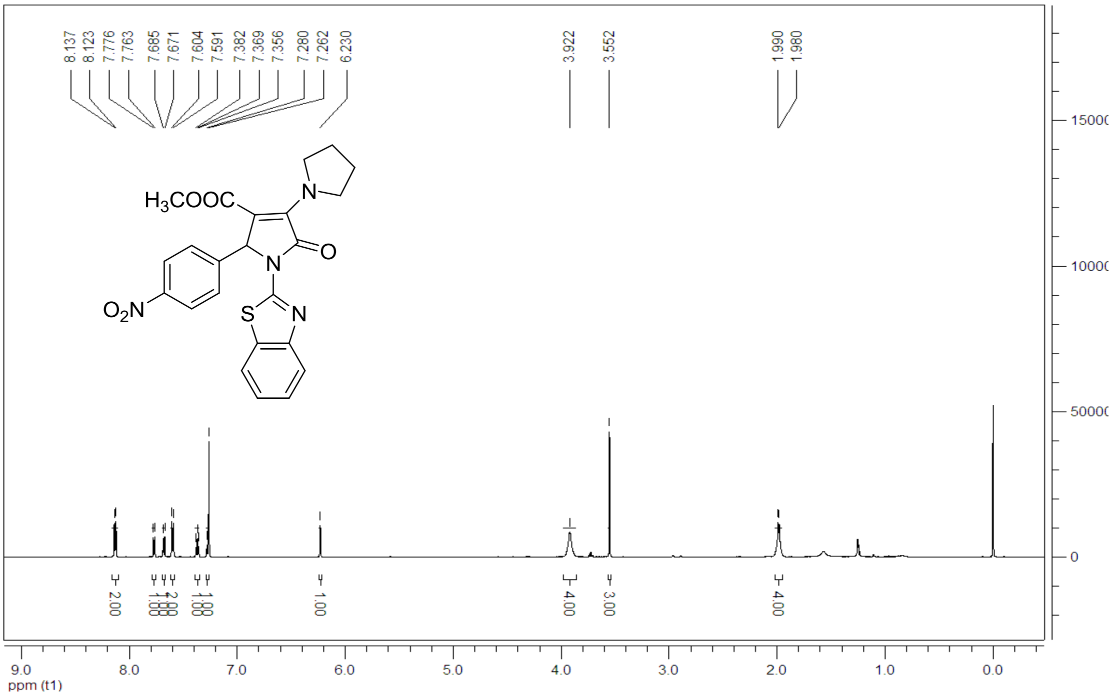

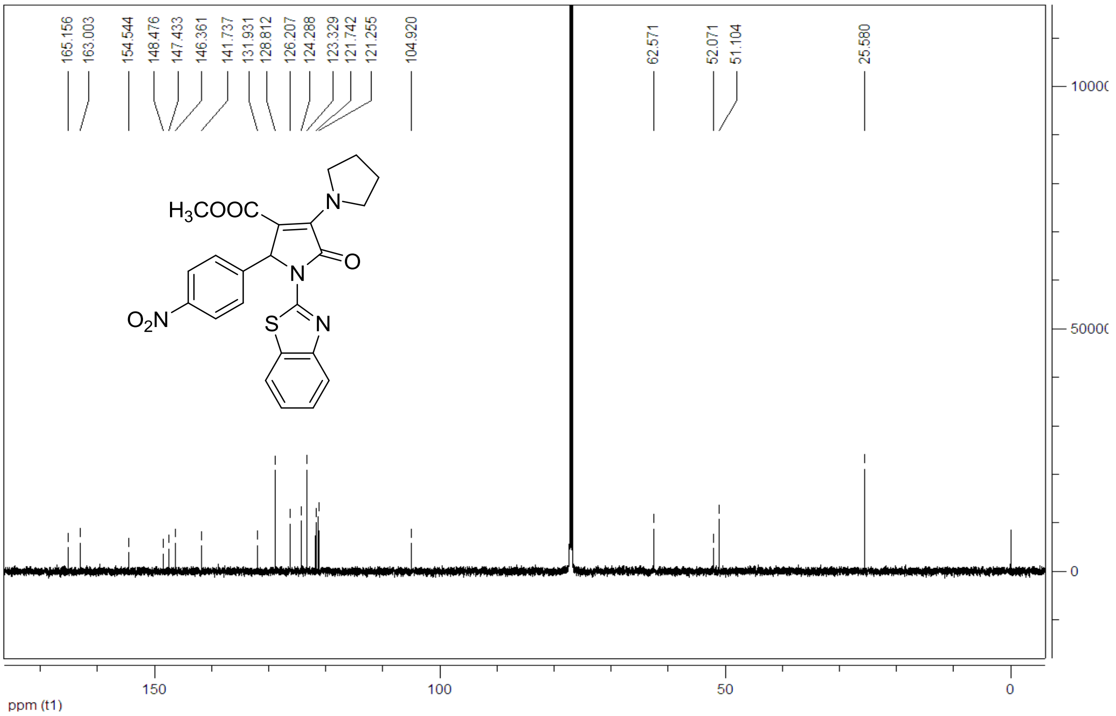

Methyl 1-(benzo[*d*]thiazol-2-yl)-2-(4-methoxyphenyl)-4-morpholino-5-oxo-2,5-dihydro-1*H*-pyrrole-3-carboxylate (1n): white solid, 10%, m.p.213.4~213.7℃; ^{1}H NMR (600 MHz, $CDCl_3$) δ: 7.75 (d, *J* = 10.2Hz, 2H, ArH), 7.37~7.26 (m, 4H, ArH), 6.79 (s, 2H, ArH), 6.13 (s, 1H, CH), 3.87 (s, 6H, CH_2), 3.74 (s, 5H, OCH_3, CH_2), 3.60 (s, 3H, OCH_3); ^{13}C NMR (150 MHz, $CDCl_3$) δ: 165.8, 163.1, 159.2, 156.8, 154.6, 154.5, 148.8, 143.7, 132.1, 129.0, 126.0, 124.1, 121.9, 121.2, 113.5, 67.4, 62.6, 55.1, 51.4, 50.2; IR (KBr) υ: 3695, 3303, 3063, 3021, 2958, 2903, 2847, 1893, 1695, 1608, 1511, 1443, 1374, 1282, 1180, 1118, 1089, 1032, 940, 873, 836, 756cm^{-1}; HRMS (ESI) Calcd. for $C_{24}H_{23}N_3NaO_5S$ ($[M+Na]^+$): 488.1251. Found: 488.1241.

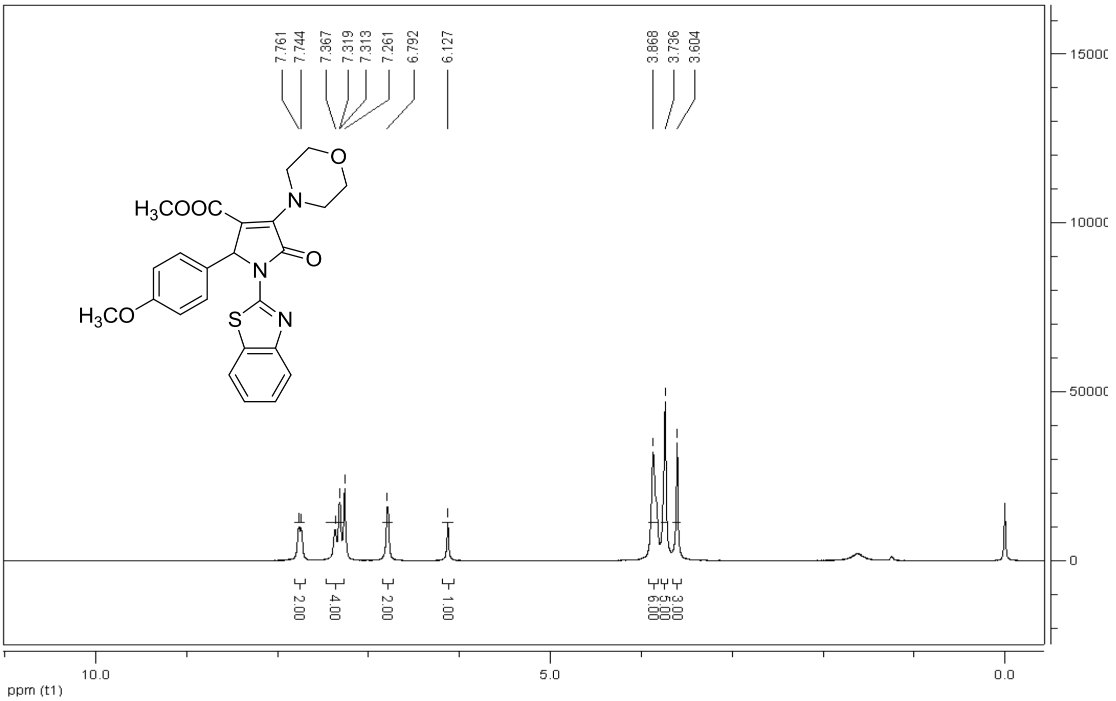

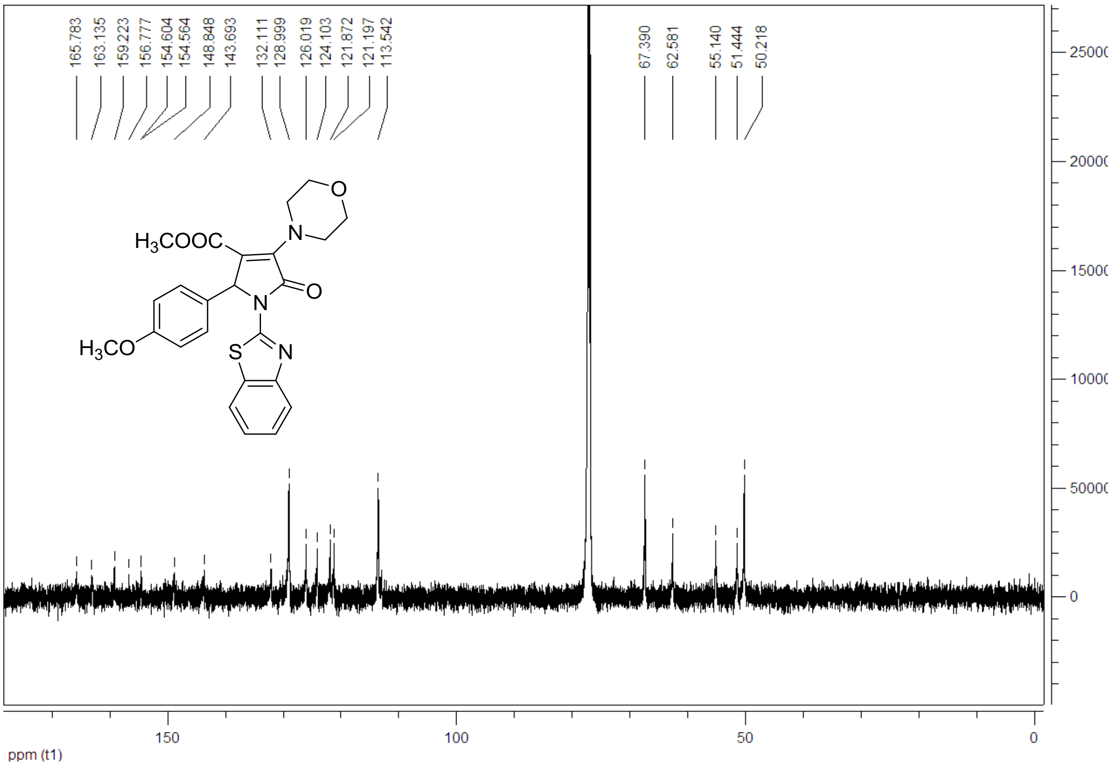

Morpholinium 1-benzothiazol-2-yl-4-methoxycarbonyl-2-oxo-5-(4-methoxyphenyl)-2,5-dihydro-1*H*-pyrrol-3-olate (2a): white solid, 75%, m.p. 192.1~192.5℃; ^{1}H NMR (600 MHz, DMSO) δ: 9.88~9.84 (m, 2H, NH), 7.93 (d, J = 7.8Hz, 1H, ArH), 7.64 (d, J = 7.8Hz, 1H, ArH), 7.37 (t, J = 7.8Hz, 1H, ArH), 7.27 (t, J = 7.2Hz, 3H, ArH), 6.79 (d, J = 8.4Hz, 2H, ArH), 5.90 (s, 1H, CH), 3.83 (s, 4H, CH_2), 3.66 (s, 3H, OCH_3), 3.48 (s, 3H, OCH_3), 3.16 (s, 4H, CH_2); ^{13}C NMR (150 MHz, DMSO) δ: 168.6, 166.4, 164.2, 158.2, 155.0, 148.5, 131.8, 131.3, 128.5, 126.0, 125.4, 123.7, 121.7, 121.0, 120.8, 120.7, 117.7, 113.0, 105.1, 63.4, 60.4, 54.8, 49.8, 42.9; IR (KBr) υ: 3411, 3070, 2966, 2907, 2840, 2628, 2458, 1717, 1674, 1617, 1552, 1514, 1448, 1361, 1277, 1227, 1197, 1139, 1108, 1079, 1026, 927, 876, 845, 757cm^{-1}; HRMS (ESI) Calcd. for $C_{20}H_{15}N_2O_5S$ [M-$C_4H_{10}NO$]: 395.0707. Found: 395.0706.

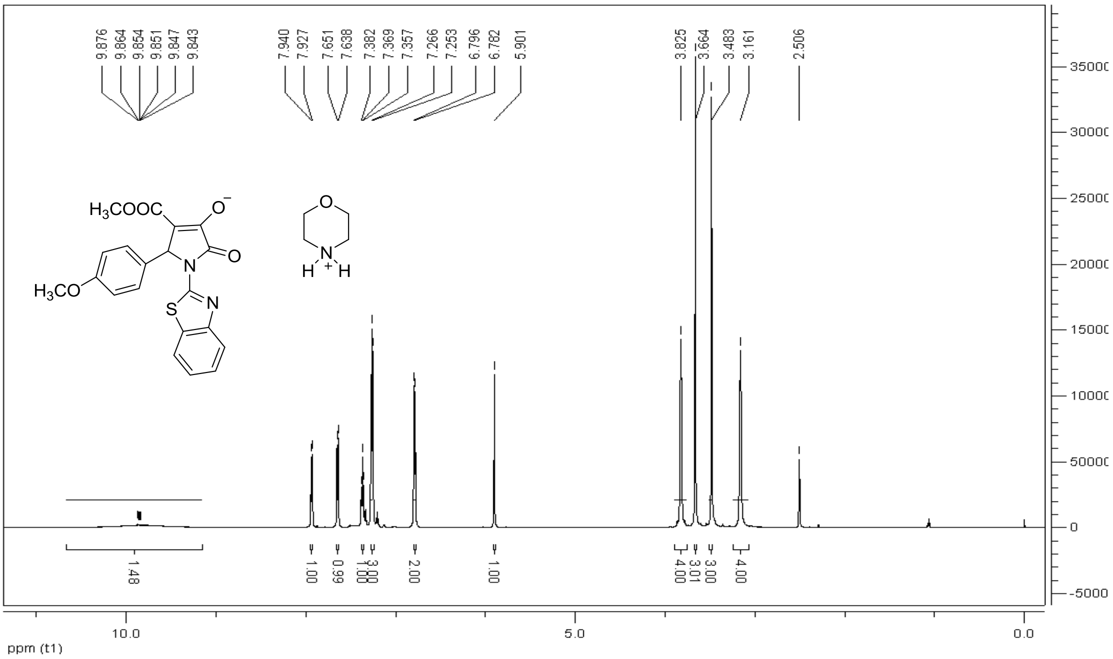

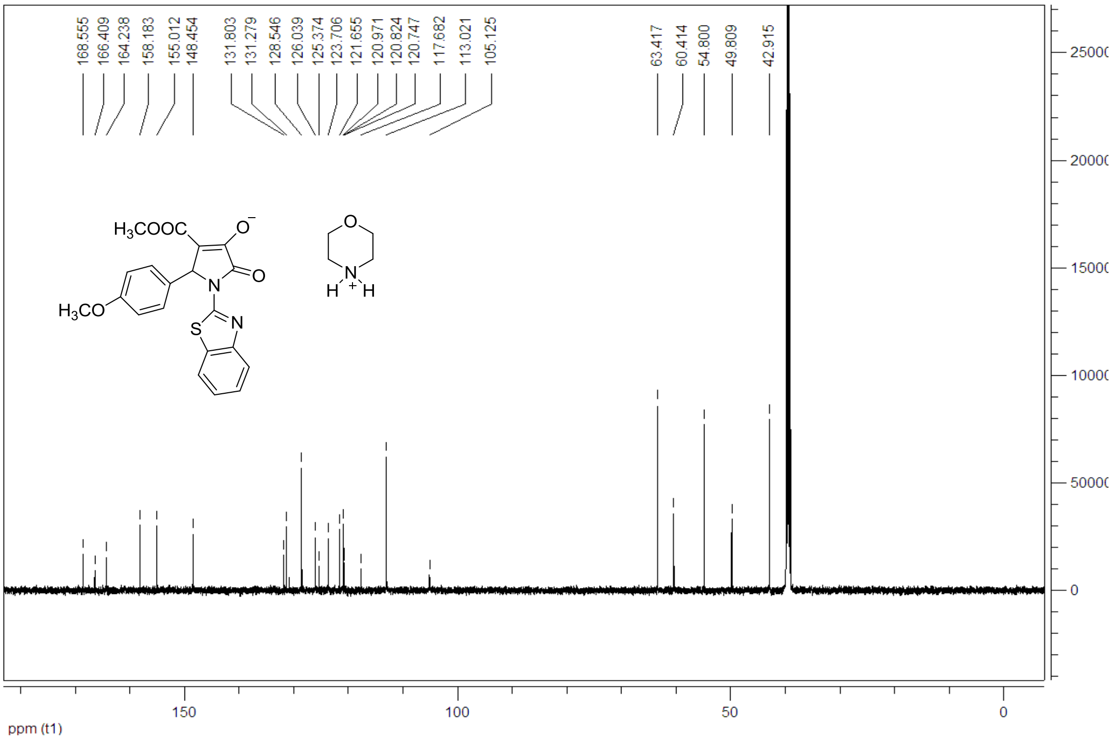

Morpholinium 1-benzothiazol-2-yl-4-methoxycarbonyl-2-oxo-5-(4-isopropylphenyl)-2,5-dihydro-1*H*-pyrrol-3-olate (2b): white solid, 73%, m.p. 196.2~196.5℃; ^{1}H NMR (600 MHz, $CDCl_3$) δ: 7.74 (d, *J* = 7.2Hz, 2H, ArH), 7.35 (t, *J* = 7.2Hz, 1H, ArH), 7.32 (d, *J* = 7.2Hz, 2H, ArH), 7.23 (t, *J* = 7.2Hz, 1H, ArH), 7.08 (d, *J* = 7.2Hz, 2H, ArH), 6.12 (s, 1H, CH), 3.94 (s, 4H, CH_2), 3.58 (s, 3H, OCH_3), 3.40 (s, 4H, CH_2), 2.82 (t, *J* = 6.6Hz, 1H, CH), 1.18 (d, *J* = 6.6Hz, 6H, CH_3); ^{13}C NMR (150 MHz, $CDCl_3$) δ: 169.4, 165.8, 163.7, 155.3, 149.2, 147.7, 136.4, 132.3, 127.5, 125.9, 125.8, 123.7, 121.8, 121.1, 106.9, 64.2, 61.2, 50.4, 43.4, 33.6, 23.9, 23.8; IR (KBr) υ: 3391, 3055, 2957, 2864, 2742, 2658, 2479, 2221, 1906, 1700, 1572, 1513, 1448, 1373, 1315, 1278, 1231, 1200, 1146, 1107, 1082, 1019, 929, 877, 845, 759cm^{-1}; HRMS (ESI) Calcd. for $C_{22}H_{19}N_2O_4S$ [M-$C_4H_{10}NO$]: 407.1071. Found: 407.1044.

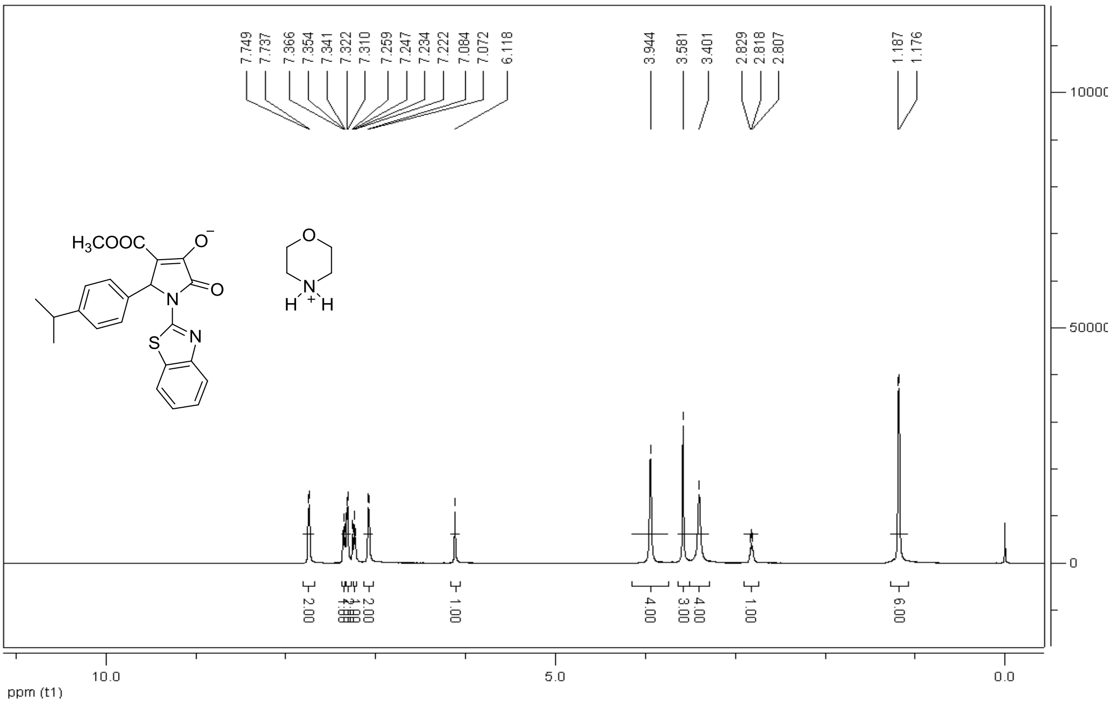

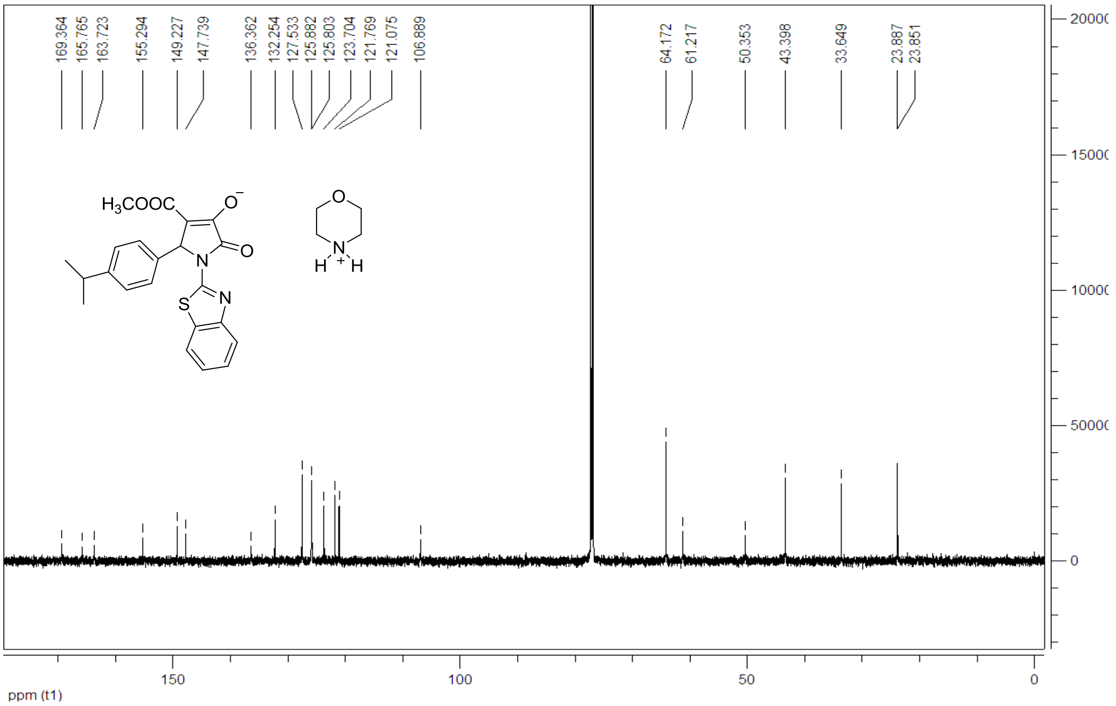

Morpholinium 1-benzothiazol-2-yl-4-methoxycarbonyl-2-oxo-5-(4-methylphenyl)-2,5-dihydro-1*H*-pyrrol-3-olate (2c): white solid, 65%, m.p. 218.3~218.7℃; ^{1}H NMR (600 MHz, $CDCl_3$) δ: 7.73 (t, *J* = 7.8Hz, 2H, ArH), 7.34 (t, *J* = 7.8Hz, 1H, ArH), 7.30 (s, 2H, ArH), 7.23 (t, *J* = 7.8Hz, 1H, ArH), 7.04 (s, 2H, ArH), 6.10 (s, 1H, CH), 3.95 (s, 4H, CH_2), 3.57 (s, 3H, OCH_3), 3.40 (s, 4H, CH_2), 2.26 (s, 3H, CH_3); ^{13}C NMR (150 MHz, $CDCl_3$) δ: 169.6, 166.0, 164.3, 155.3, 149.2, 136.9, 136.2, 132.3, 128.6, 127.6, 125.8, 123.7, 121.8, 121.0, 106.5, 64.4, 61.3, 50.3, 43.6, 21.0; IR (KBr) υ: 3543, 3054, 2849, 2650, 2458, 1708, 1678, 1580, 1516, 1446, 1365, 1318, 1278, 1227, 1201, 1110, 1082, 1042, 1018, 930, 879, 847, 824, 757cm^{-1}; HRMS (ESI) Calcd. for $C_{20}H_{15}N_2O_4S$ [M-$C_4H_{10}NO$]: 379.0718. Found: 379.0720.

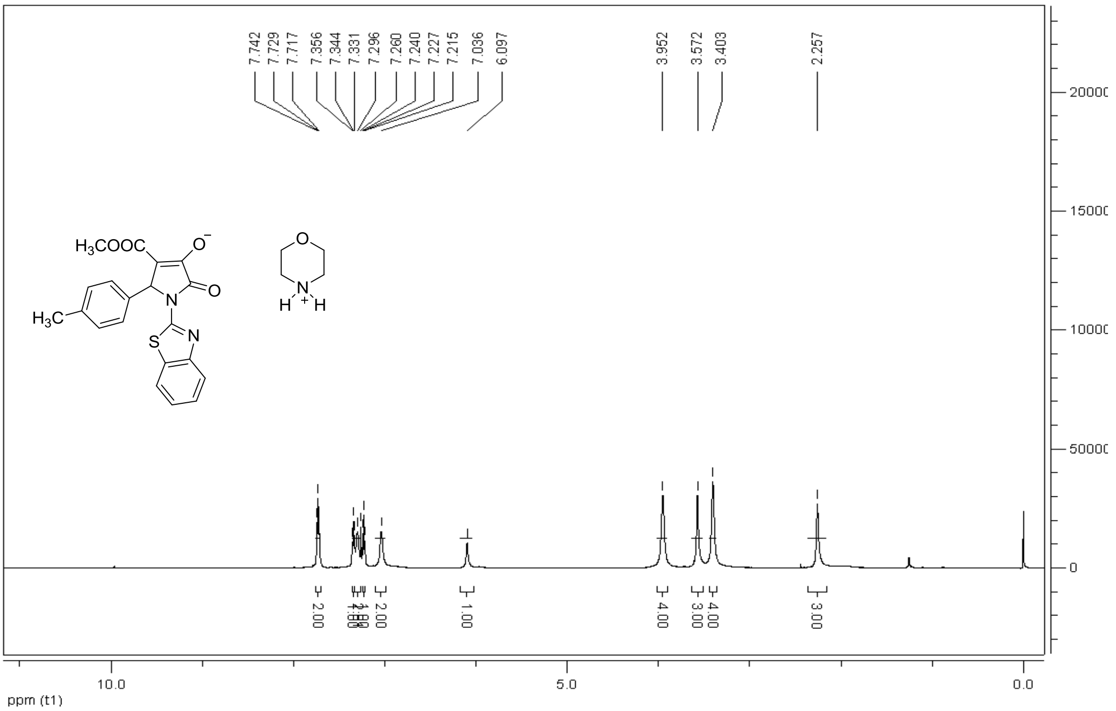

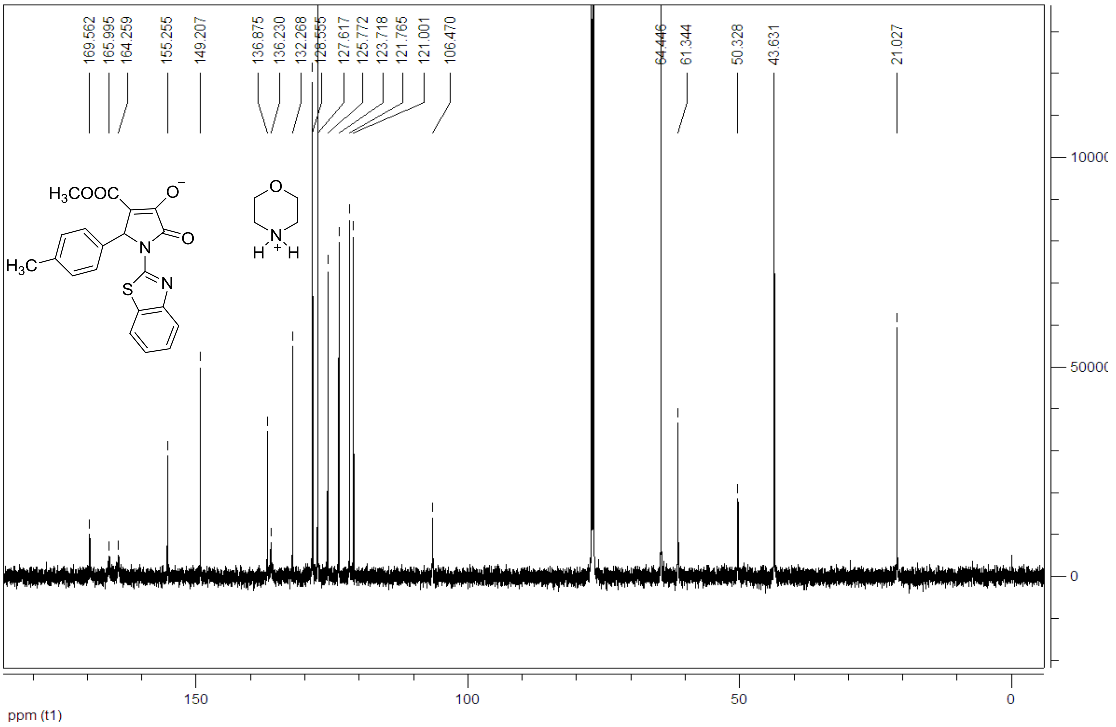

Morpholinium 1-benzothiazol-2-yl-4-methoxycarbonyl-2-oxo-5-phenyl-2,5-dihydro-1*H*-pyrrol-3-olate (2d): white solid, 87%, m.p. 221.3~221.6℃; ^{1}H NMR (600 MHz, DMSO) δ: 9.84~9.79 (m, 2H, NH), 7.92 (d, *J* = 7.8Hz, 1H, ArH), 7.62 (d, *J* = 7.8Hz, 1H, ArH), 7.36~7.32 (m, 3H, ArH), 7.27~7.21 (m, 3H, ArH), 7.13 (t, *J* = 6.6Hz, 1H, ArH), 5.92 (s, 1H, CH), 3.83 (s, 4H, CH_2), 3.46 (s, 3H, OCH_3), 3.16 (s, 4H, CH_2); ^{13}C NMR (150 MHz, DMSO) δ: 169.3, 164.5, 162.6, 155.2, 148.4, 140.8, 131.3, 127.6, 127.3, 126.9, 126.0, 123.6, 121.6, 120.9, 103.6, 63.4, 60.8, 49.5, 42.9; IR (KBr) υ: 3586, 3519, 3080, 2971, 2868, 2712, 2459, 1685, 1614, 1570, 1446, 1366, 1313, 1275, 1226, 1200, 1169, 1104, 1044, 928, 876, 827, 762cm^{-1}; HRMS (ESI) Calcd. for $C_{19}H_{13}N_2O_4S$ [M-$C_4H_{10}NO$]: 365.0602. Found: 365.0596

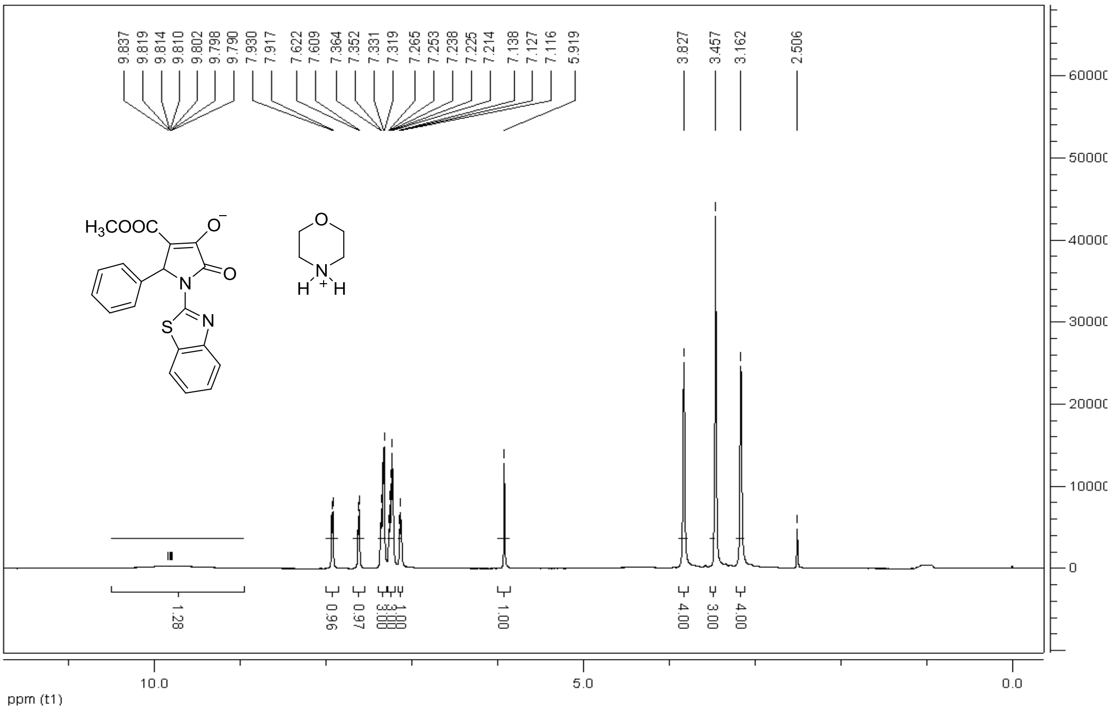

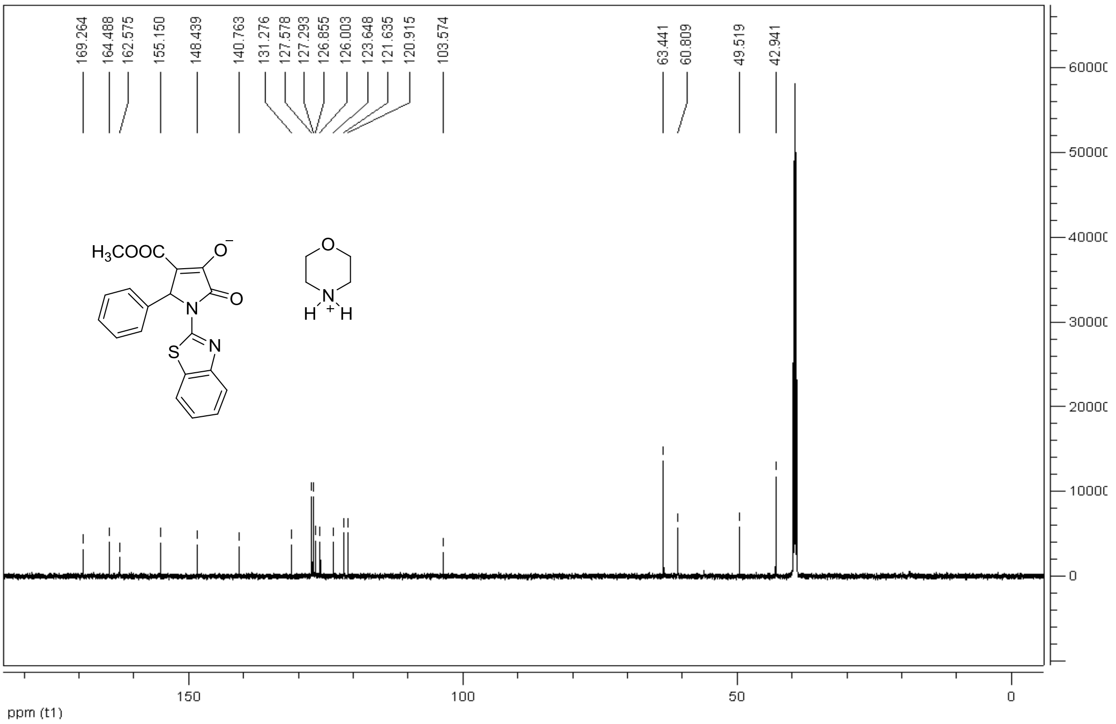

Morpholinium 1-benzothiazol-2-yl-4-methoxycarbonyl-2-oxo-5-(3-nitrophenyl)-2,5-dihydro-1*H*-pyrrol-3-olate (2e): white solid, 79%, m.p. 197.7~197.9℃; ^{1}H NMR (600 MHz, DMSO) δ: 9.62~9.60 (m, 2H, NH), 8.16 (s, 1H, ArH), 8.05 (d, *J* = 7.8Hz, 1H, ArH), 7.94 (d, *J* = 7.2Hz, 1H, ArH), 7.82 (d, *J* = 7.8Hz, 1H, ArH), 7.60~7.55 (m, 2H, ArH), 7.36 (t, *J* = 7.8Hz, 1H, ArH), 7.27 (t, *J* = 7.8Hz, 1H, ArH), 6.04 (s, 1H, CH), 3.83 (s, 4H, CH_2), 3.46 (s, 3H, OCH_3), 3.17 (s, 4H, CH_2); ^{13}C NMR (150 MHz, DMSO) δ: 169.0, 164.4, 162.7, 155.1, 148.2, 147.2, 143.5, 134.3, 131.2, 129.2, 126.1, 123.8, 122.1, 122.0, 121.7, 120.9, 102.5, 63.4, 60.2, 49.7, 42.9; IR (KBr) υ: 3401, 3055, 2970, 2856, 2622, 2408, 1709, 1681, 1581, 1523, 1446, 1357, 1282, 1253, 1228, 1201, 1166, 1093, 1025, 930, 880, 826, 754cm^{-1}; HRMS (ESI) Calcd. for $C_{19}H_{12}N_3O_6S$ [M-C_4H_{10}NO]: 410.0452. Found: 410.0443.

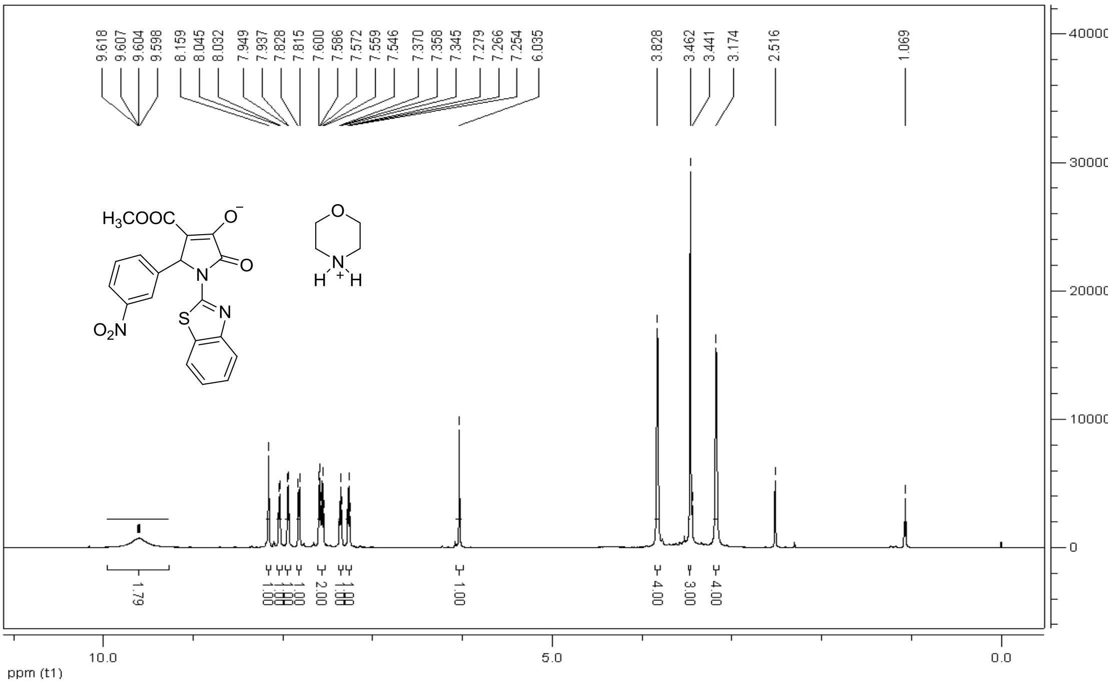

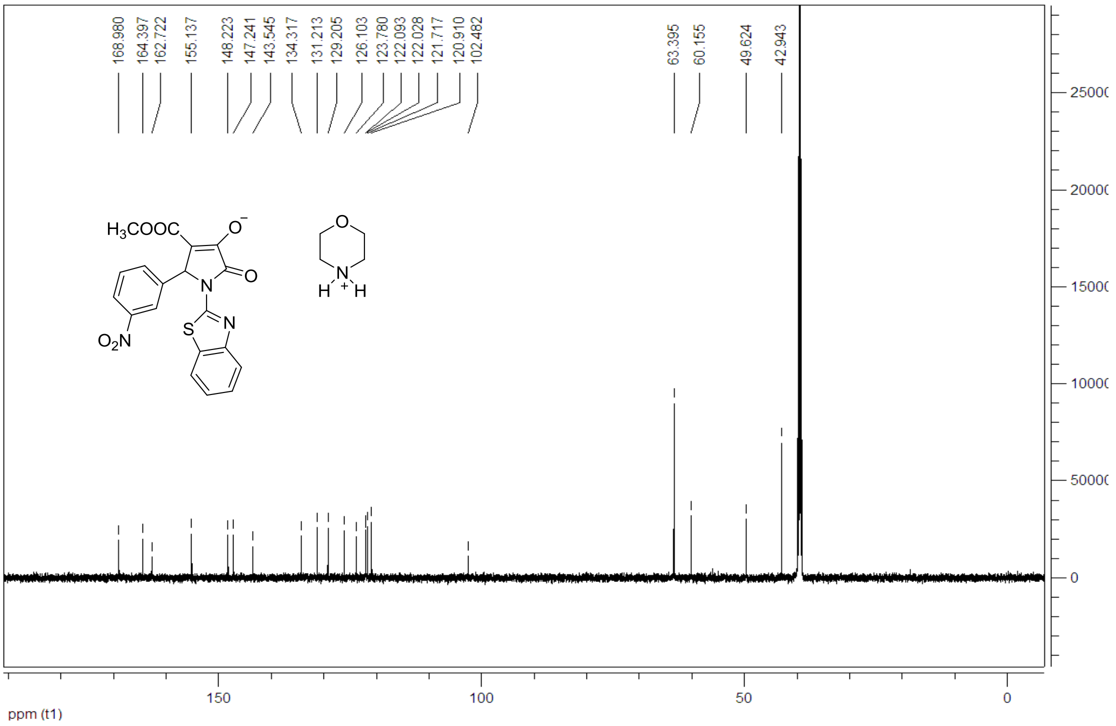

Piperidinium 1-benzothiazol-2-yl-4-methoxycarbonyl-2-oxo-5-(4-methylphenyl)-2,5-dihydro-1*H*-pyrrol-3-olate (2f): white solid, 75%, m.p. 198~200℃; ^{1}H NMR (600 MHz, $CDCl_3$) δ: 9.75~9.73 (m, 2H, NH), 7.72 (d, J = 7.8Hz, 2H, ArH), 7.33 (t, J = 7.8Hz, 3H, ArH), 7.20 (t, J = 7.8Hz, 1H, ArH), 7.02 (d, J = 7.2Hz, 2H, ArH), 6.09 (s, 1H, CH), 3.56 (s, 3H, OCH_3), 3.28 (s, 4H, CH_2), 2.25 (s, 3H, CH_3), 1.77 (s, 4H, CH_2), 1.63 (s, 2H, CH_2); ^{13}C NMR (150 MHz, DMSO-d_6) δ: 169.5, 164.6, 163.0, 155.2, 148.5, 138.0, 135.8, 131.3, 128.2, 127.2, 126.0, 123.6, 121.6, 120.9, 102.8, 60.5, 49.4, 43.8, 22.2, 21.9, 20.6; IR (KBr) υ: 3445, 3057, 2948, 2861, 2721, 2628, 2534, 2438, 1699, 1618, 1575, 1515, 1441, 1364, 1276, 1228, 1194, 1083, 1019, 928, 838, 759cm^{-1}; HRMS (ESI) Calcd. for $C_{20}H_{15}N_2O_4S$ [M-$C_5H_{12}N$]: 379.0758. Found: 379.0755.

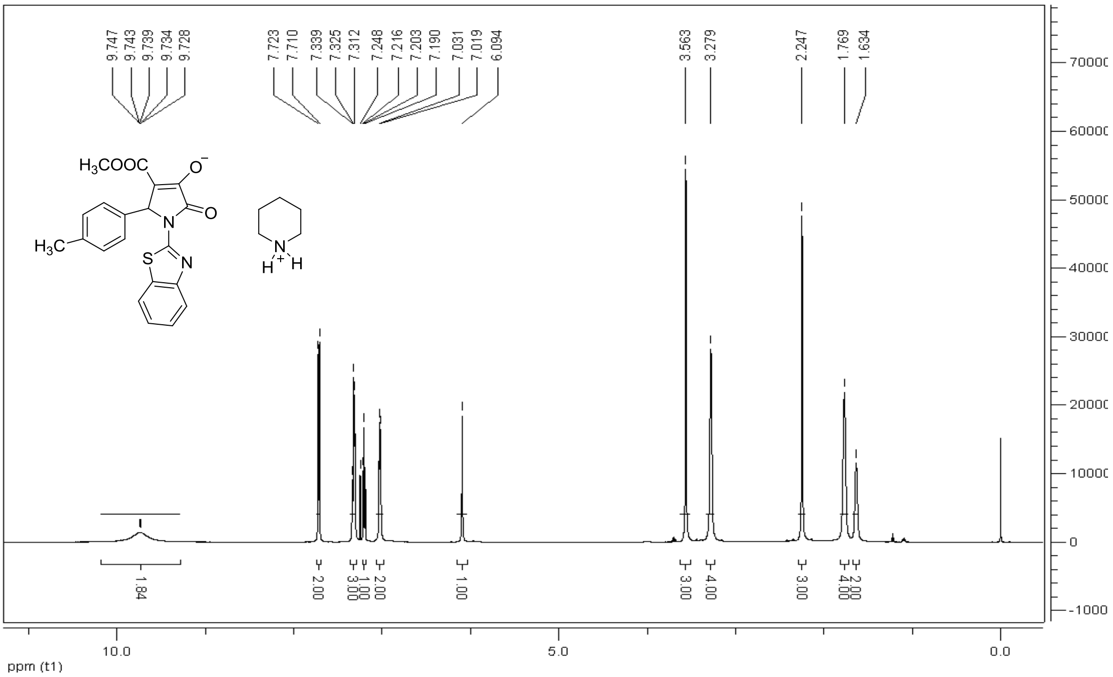

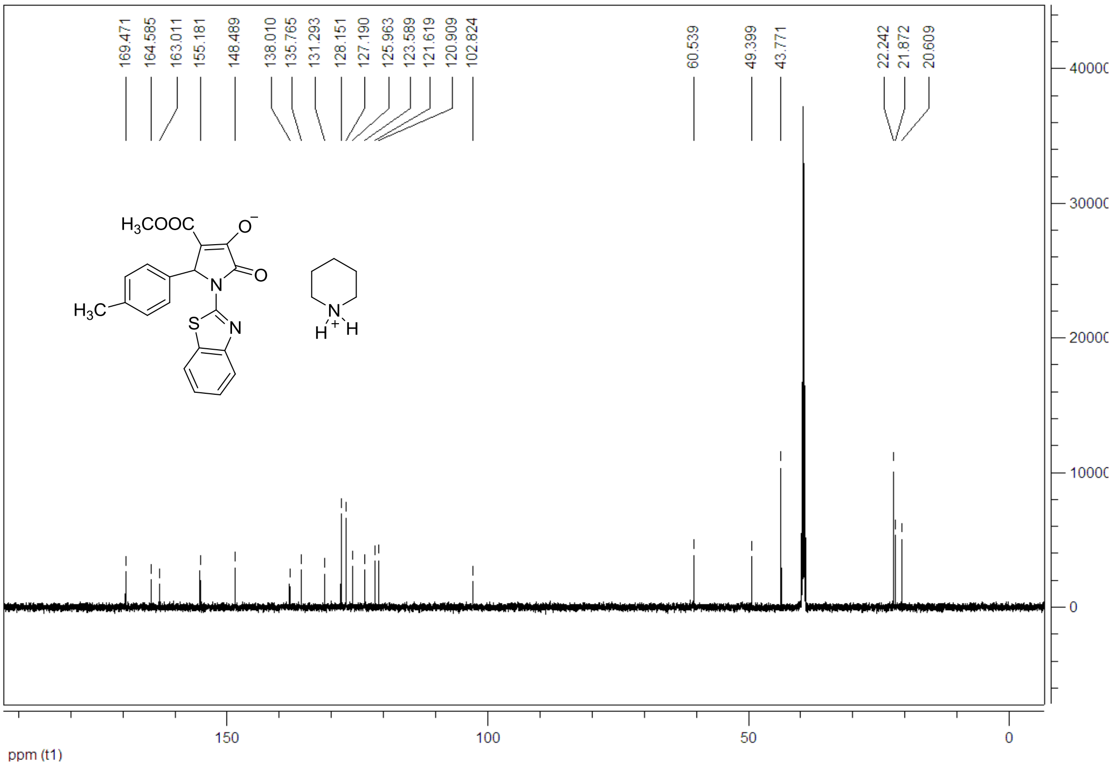

Piperidinium 1-benzothiazol-2-yl-4-methoxycarbonyl-2-oxo-5-(3-methylphenyl)-2,5-dihydro-1*H*-pyrrol-3-olate (2g): light red solid, 72%, m.p. 210~212℃; ^{1}H NMR (600 MHz, DMSO-d_6) δ: 9.08 (s, 2H, NH), 7.93 (d, *J* = 7.8Hz, 1H, ArH), 7.62 (d, *J* = 7.8Hz, 1H, ArH), 7.36 (t, *J* = 7.8Hz, 1H, ArH), 7.26 (t, *J* = 7.2Hz, 1H, ArH), 7.15 (d, *J* = 7.2Hz, 1H, ArH), 7.11 (t, *J* = 7.8Hz, 1H, ArH), 7.06 (s, 1H, ArH), 6.93 (d, *J* = 7.2Hz, 1H, ArH), 5.85 (s, 1H, CH), 3.44 (s, 3H, OCH_3), 3.05 (s, 4H, CH_2), 2.21 (s, 3H, CH_3), 1.68 (s, 4H, CH_2), 1.57 (s, 2H, CH_2); ^{13}C NMR (150 MHz, DMSO-d_6) δ: 169.0, 164.4, 161.9, 161.8, 155.1, 148.5, 140.5, 136.5, 131.3, 127.7, 127.4, 127.3, 126.0, 125.1, 123.7, 121.6, 121.0, 104.2, 60.8, 49.6, 43.8, 22.2, 21.9, 21.0; IR (KBr) υ: 3420, 2945, 2861, 2731, 2549, 1705, 1588, 1519, 1439, 1361, 1322, 1274, 1230, 1195, 1136, 1084, 1019, 930, 871, 757cm^{-1}; HRMS (ESI) Calcd. for $C_{20}H_{15}N_2O_4S$ [M-$C_5H_{12}N$]: 379.0758. Found: 379.0760.

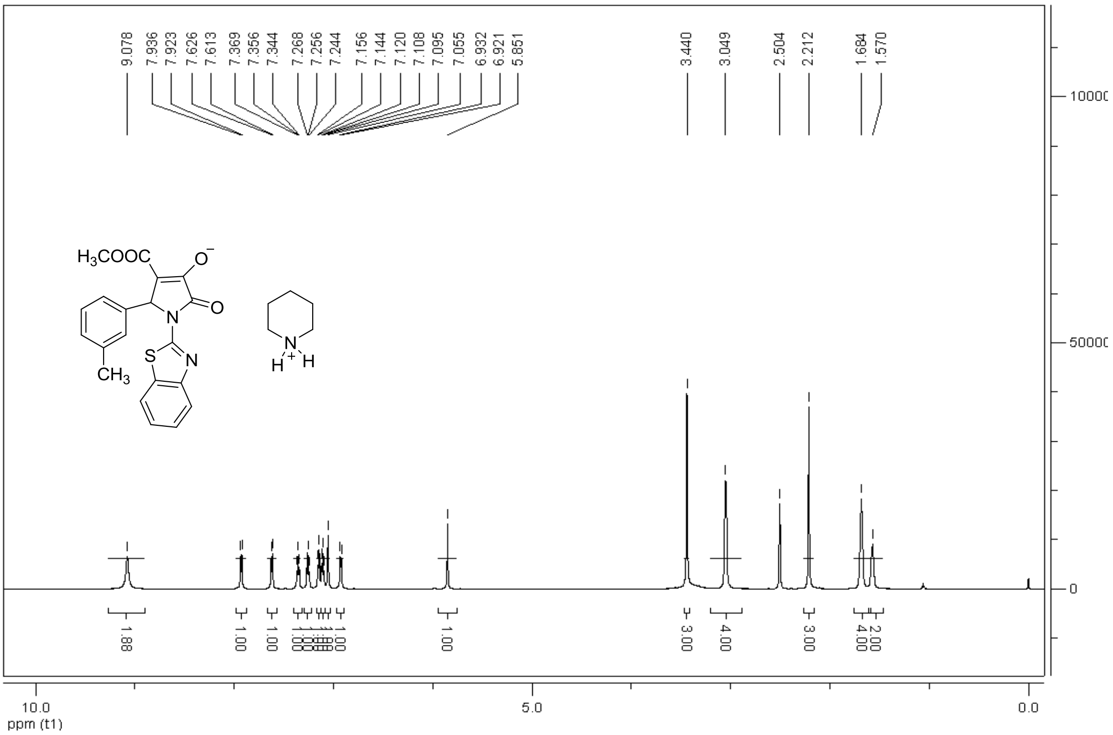

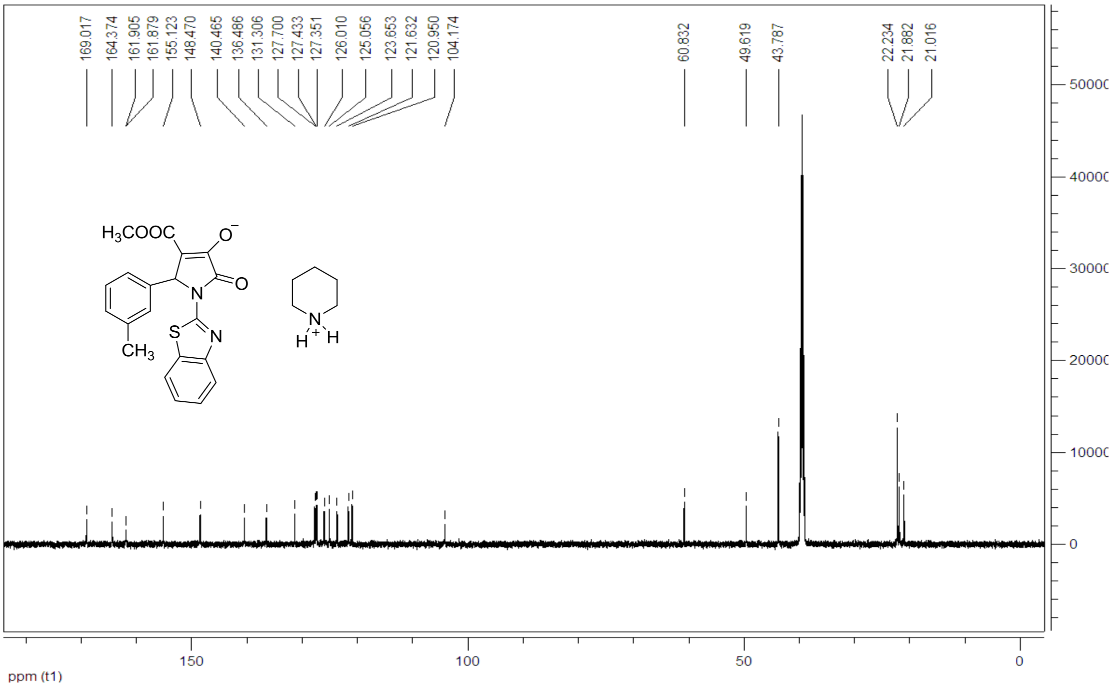

Piperidinium 1-benzothiazol-2-yl-4-methoxycarbonyl-2-oxo-5-(4-*tert*-butylphenyl)-2,5-dihydro-1*H*-pyrrol-3-olate (2h): white solid, 80%, m.p. 209~211℃; ^{1}H NMR (600 MHz, $CDCl_3$) δ: 9.77~9.75 (m, 2H, NH), 7.75~7.73 (m, 2H, ArH), 7.35~7.32 (m, 3H, ArH), 7.22 (t, J = 7.2Hz, 3H, ArH), 6.12 (s, 1H, CH), 3.57 (s, 3H, OCH_3), 3.27 (s, 4H, CH_2), 1.76 (s, 4H, CH_2), 1.63 (s, 2H, CH_2), 1.24 (s, 9H, CH_3); ^{13}C NMR (150 MHz, DMSO-d_6) δ: 169.6, 164.6, 162.9, 155.3, 148.8, 148.5, 138.0, 131.3, 126.9, 126.0, 124.4, 123.6, 121.6, 120.9, 102.8, 60.3, 49.4, 43.8, 34.1, 31.1, 22.2, 21.9; IR (KBr) υ: 3445, 3063, 2957, 2864, 2730, 2656, 2540, 2362, 1701, 1624, 1558, 1516, 1441, 1368, 1362, 1274, 1229, 1197, 1145, 1118, 1082, 1019, 930, 843, 759cm^{-1}; HRMS (ESI) Calcd. for $C_{23}H_{21}N_2O_4S$ [M-$C_5H_{12}N$]: 421.1228. Found: 421.1228.

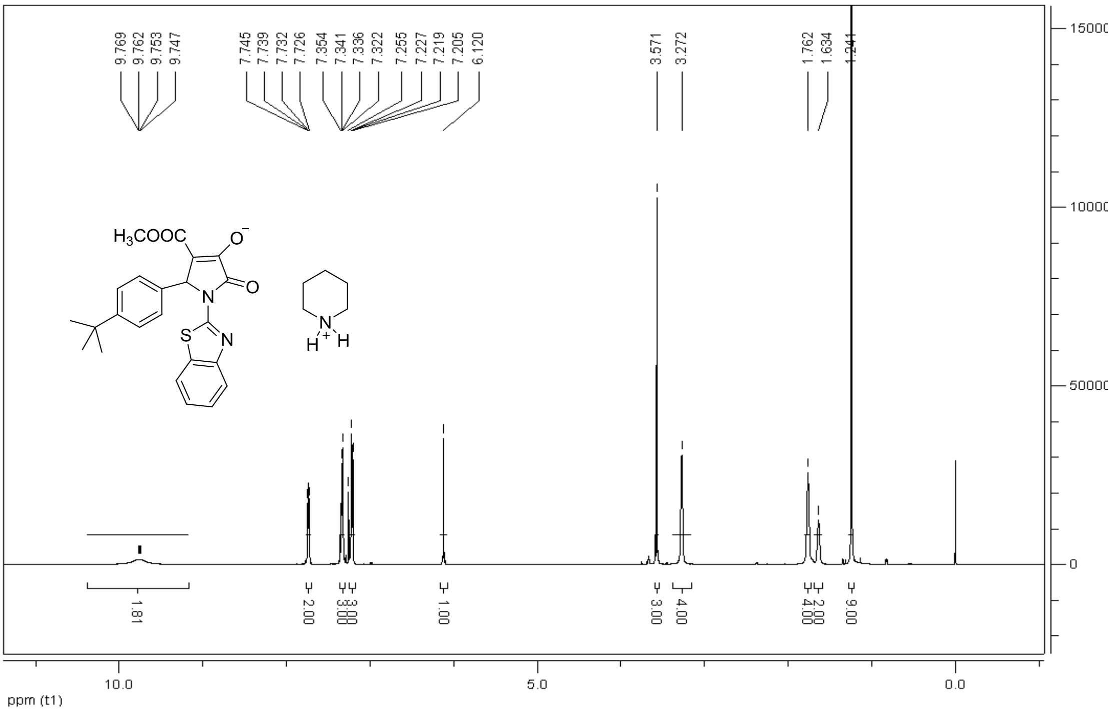

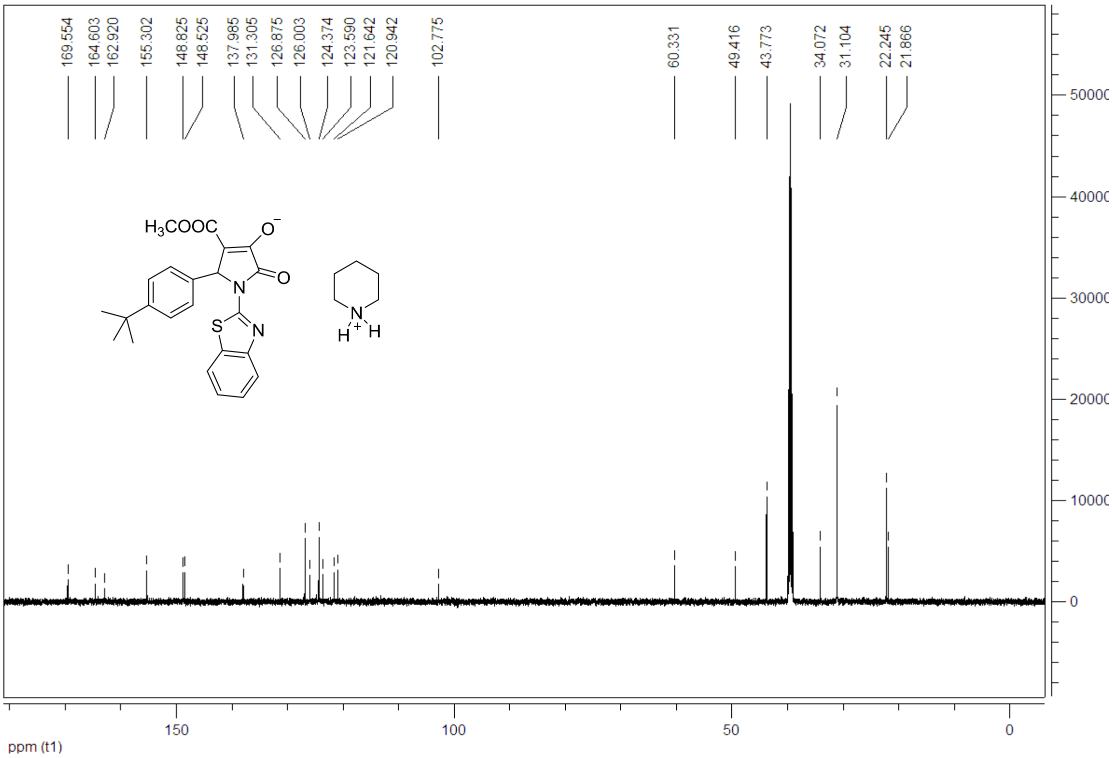

Supplement: File 1 — Analytical data and 1H and 13C NMR spectra. [file Beilstein_J_Org_Chem-09-2934-s001.pdf]
